# Pursuing personalized medicine for depression by targeting the lateral or medial prefrontal cortex with Deep TMS

**DOI:** 10.1172/jci.insight.165271

**Published:** 2023-02-22

**Authors:** Abraham Zangen, Samuel Zibman, Aron Tendler, Noam Barnea-Ygael, Uri Alyagon, Daniel M. Blumberger, Geoffrey Grammer, Hadar Shalev, Tatiana Gulevski, Tanya Vapnik, Alexander Bystritsky, Igor Filipčić, David Feifel, Ahava Stein, Frederic Deutsch, Yiftach Roth, Mark S. George

**Affiliations:** 1 Ben-Gurion University of the Negev, Beer-Sheva, Israel.; 2 Advanced Mental Health Care Inc., Royal Palm Beach, Florida, USA.; 3 Temerty Centre for Therapeutic Brain Intervention, Centre for Addiction and Mental Health, and Department of Psychiatry, University of Toronto, Toronto, Ontario, Canada.; 4 Greenbrook TMS NeuroHealth, McLean, Virginia, USA.; 5 Department of Psychiatry, Soroka Medical Center, Beer-Sheva, Israel.; 6 Pacific Institute of Medical Research, Los Angeles, California, USA.; 7 Psychiatric Hospital Sveti Ivan and School of Medicine, University of Zagreb, Zagreb, Croatia.; 8 Kadima Neuropsychiatry Institute, La Jolla, California, USA.; 9 A. Stein – Regulatory Affairs Consulting Ltd, Kfar Saba, Israel.; 10 BioStats Statistical Consulting Ltd., Modiin, Israel.; 11 Medical University of South Carolina, Columbia, South Carolina, USA.; 12 Ralph H. Johnson VA Medical Center, Charleston, South Carolina, USA.

**Keywords:** Clinical Trials, Neuroscience, Behavior, Depression, Neuroimaging

## Abstract

**BACKGROUND:**

Major depressive disorder (MDD) can benefit from novel interventions and personalization. Deep transcranial magnetic stimulation (Deep TMS) targeting the lateral prefrontal cortex (LPFC) using the H1 coil was FDA cleared for treatment of MDD. However, recent preliminary data indicate that targeting the medial prefrontal cortex (MPFC) using the H7 coil might induce outcomes that are as good or even better. Here, we explored whether Deep TMS targeting the MPFC is noninferior to targeting the LPFC and whether electrophysiological or clinical markers for patient selection can be identified.

**METHODS:**

The present prospective, multicenter, randomized study enrolled 169 patients with MDD for whom antidepressants failed in the current episode. Patients were randomized to receive 24 Deep TMS sessions over 6 weeks, using either the H1 coil or the H7 coil. The primary efficacy endpoint was the change from baseline to week 6 in Hamilton Depression Rating Scale scores.

**RESULTS:**

Clinical efficacy and safety profiles were similar and not significantly different between groups, with response rates of 60.9% for the H1 coil and 64.2% for the H7 coil. Moreover, brain activity measured by EEG during the first treatment session correlated with clinical outcomes in a coil-specific manner, and a cluster of baseline clinical symptoms was found to potentially distinguish between patients who can benefit from each Deep TMS target.

**CONCLUSION:**

This study provides a treatment option for MDD, using the H7 coil, and initial guidance to differentiate between patients likely to respond to LPFC versus MPFC stimulation targets, which require further validation studies.

**TRIAL REGISTRATION:**

ClinicalTrials.gov NCT03012724.

**FUNDING:**

BrainsWay Ltd.

## Graphical abstract

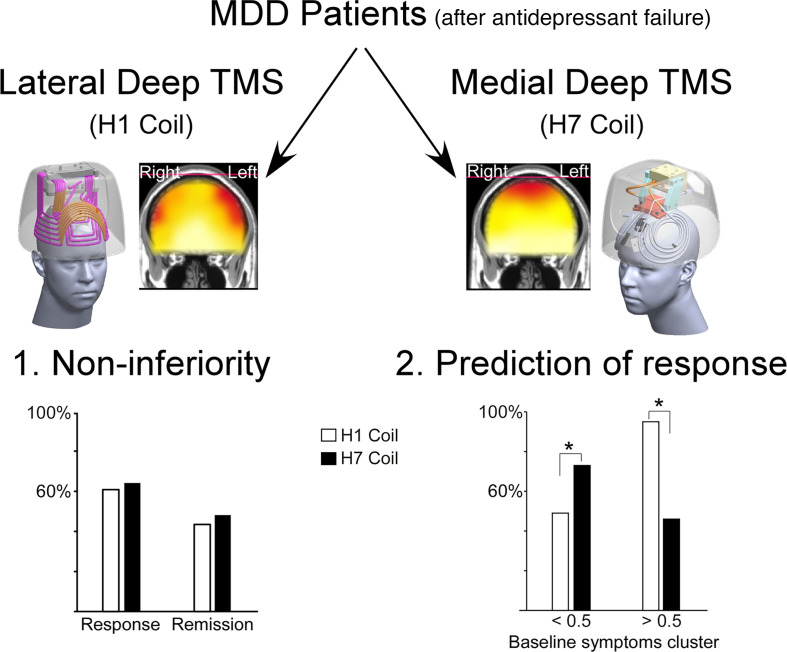

## Introduction

Depression is among the leading causes of disability, negatively influences quality of life, and is the main determinant of suicide (1, 2). The impact and prevalence of depression worsened during the COVID-19 pandemic (3), and the majority of people experiencing depression still do not receive adequate treatment (1). Nonresponse to treatment is partly related to the heterogeneous nature of depression, encompassing several subsyndromes characterized by distinct dysfunctional patterns of brain activity (4). However, due to the lack of a priori markers for personalized treatment, the trial-and-error approach is typically used for pharmacological or other antidepressant treatments (5).

Transcranial magnetic stimulation (TMS) is a safe and well-tolerated intervention that has been extensively studied as a treatment for major depressive disorder (MDD), with over 150 randomized controlled trials (RCTs) and confirmed efficacy in many meta-analyses (6). Notably, TMS directed to the lateral prefrontal cortex (LPFC) is accepted as a treatment option in refractory depression, with both traditional TMS that uses a figure eight coil and Deep TMS that uses H1 coil protocols granted FDA clearance following large multicenter RCTs (7, 8). Following these treatments, 25%–35% of patients with medication-resistant depression remit (largely symptom free), and an additional 15%–25% respond (symptoms decrease by more than 50%) (6). Here again, a priori markers for personalized medicine have yet to be identified, and the established treatment paradigms of LPFC stimulation may have inadvertently distracted from searching for alternative, effective targets (9).

The LPFC is only 1 target identified in a common brain circuit of effective neurostimulation for depression (10), and different targets may be more effective in treating patients with different subsyndromes (11) that correspond to distinct dysfunctional patterns of brain activity (4). Thus, stimulation protocols targeting other brain areas may be beneficial for certain patients.

Recently, the medial prefrontal cortex (MPFC) along with the anterior cingulate cortex (ACC) were posited as promising alternative targets for Deep TMS treatment of MDD, due to their involvement in reward, emotions, mood, and habits (9, 12, 13). There are known direct excitatory glutamatergic projections from the ACC to the ventral striatum, and a recent study found negative associations between reward-related MPFC-striatum connectivity and increased depressive symptom severity (14). In addition, the MPFC and ACC are the most consistent regions to show gray matter reduction in patients with MDD compared with healthy controls (15). Initial evidence for the efficacy of stimulation over the MPFC was obtained in several studies that did not include a sham control arm (16–19). In these studies, overall 482 patients with MDD were treated with a D-B80 coil (an angled figure eight coil) for traditional TMS, and the weighted average response and remission rates were 41.4% and 31.5%, respectively. A sham-controlled study (20) found a significant effect in favor of the D-B80 coil (*n* = 13) versus figure eight coil (*n* = 15), but no significant effects for the D-B80 versus sham stimulation (*n* = 12), at the end of treatment (week 3). In addition, the MPFC and ACC are targeted by the Deep TMS H7 coil, which stimulates significantly deeper and broader brain volume relative to the D-B80 coil (21). While the H7 coil is used primarily for the treatment of obsessive-compulsive disorder (OCD) (22), recent preliminary results indicated that it can also induce significant antidepressant effects in patients with MDD for whom treatment failed with the Deep TMS H1 coil (23).

The purpose of the current multicenter RCT was to explore whether the efficacy of the H7 coil is noninferior to that of the FDA-cleared H1 coil in patients with MDD, with the eventual goal of providing clinicians multiple options for patient treatment. A successful show of noninferiority would mean clinicians could prescribe to any given patient either treatment with the same expected efficacy. In an attempt to guide clinicians to make informed choices regarding choice of treatment based on the literature and secondary analysis of this study’s results, a secondary purpose of this study was a preliminary exploration of whether an a priori clinical or electrophysiological differential predictor for TMS H-coils (C-DEPTH or E-DEPTH) for patient selection can be identified.

It is currently unknown which patients with MDD are expected to benefit from any specific TMS protocol, and in particular, which will benefit from lateral and which from MPFC stimulation (24). A widely accessible approach is to develop predictors based on patients’ clinical and demographic baseline (pretreatment) data (25, 26). For example, symptom clusters derived from individual items on rating scales have been used to differentiate between patients’ responses to pharmaceutical treatments for depression (27, 28). Some success has been made while applying this approach to differentiate between TMS protocols, with data from early response after several treatment sessions but not at the pretreatment baseline (29–32). In addition, several attempts at identifying biomarkers of pharmaceutical and TMS efficacy have been made based on neuroimaging data, primarily from functional magnetic resonance imaging and electroencephalogram (EEG) recordings (4, 11, 33–35). Although these procedures are less common in clinical settings (36), such data can inform treatment choices when available.

Optimally, C-DEPTH would be based on symptom clusters at baseline and would provide clinicians a straightforward method for differentiating between patients who are likely to respond to one stimulation target over the other. Here, exploration for such a clinical marker was informed by recent evidence of the H1 coil’s polysymptomatic effect on depression and anxiety symptoms (11, 22, 37–41). We hypothesized that patients showing high baseline scores in core symptoms of both depression and anxiety would respond better to the H1 coil. The exploration of an electrophysiological marker (using EEG; E-DEPTH) was based on recent evidence that alpha and low-gamma EEG bands during the first treatment session may represent the responsivity of the cortex to TMS and predict response to Deep TMS in patients with attention deficit hyperactivity disorder (ADHD) (42). In accordance, we expected that higher lateral and medial ratio scores would correlate with better clinical outcomes for the H1 and H7 coils, respectively. Please see Supplemental Data 1, “EEG” (supplemental material available online with this article; https://doi.org/10.1172/jci.insight.165271DS1), for further details.

## Results

### Patient enrollment and baseline characteristics.

**Figure 1:**
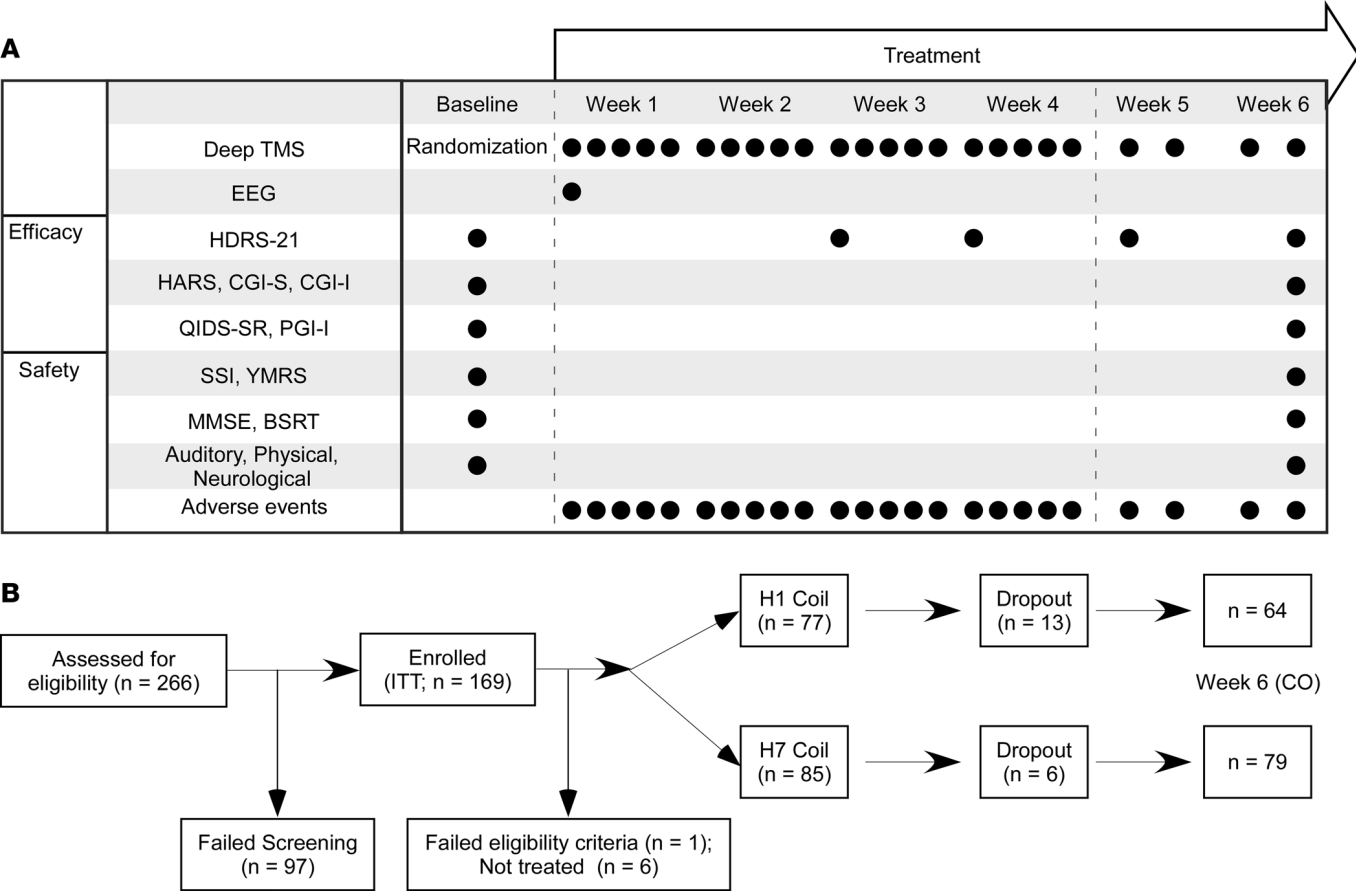
Timeline, assessments, enrollment, and randomization. (**A**) Efficacy was assessed by Hamilton Depression Rating Scale (21-item version; HDRS-21), as well as by pre- and post-treatment Hamilton Anxiety Rating Scale (HARS) and Clinical Global Impression Severity and Improvement (CGI-S and CGI-I). In addition, patients completed self-report questionnaires on a weekly basis, including the Quick Inventory of Depressive Symptoms – Self Report (QIDS-SR) and the Patient Global Impressions Improvement (PGI-I). Daily functioning score (Global Assessment of Functioning, GAF) and quality-of-life measures (Quality of Life Enjoyment and Satisfaction Questionnaire, Q-Les-Q) were collected as well. Safety was assessed at pre- and posttreatment using the Scale for Suicide Ideations (SSI), Young Manic Rating Scale (YMRS), auditory threshold tests, physical and neurological examinations, and vital signs. Additional safety assessments included cognitive changes evaluations performed throughout the study, including the Mini-Mental Status Examination (MMSE) and the Buschke Selective Reminding Test (BSRT). Throughout the entire study patients were under the direct monitoring of a physician, and any adverse effects or subjective complaints were immediately recorded and treated. (**B**) Patients were outpatients aged 22 to 68 years who signed an informed consent form and had HDRS-21 ≥ 20 that was stable between screening and baseline assessments (±30%). Main exclusion criteria included comorbid psychiatric and neurological disorders, presence of psychosis, primary anxiety disorder causing higher distress than MDD, substance abuse within 6 months, prominent personality disorder causing higher distress than MDD, dysthymia, prior head trauma or seizures, and suicide attempt within 3 years. The intent-to-treat (ITT) analysis set included all patients enrolled in the study, and the completers (CO) analysis set included all patients randomized to the study who had no major protocol violations and completed the 6 weeks of treatment.

A total of 266 MDD patients were screened and 169 enrolled in the study, which included 24 Deep TMS sessions over 6 weeks (20 sessions in the first 4 weeks and 4 sessions in the next 2 weeks; Figure 1A), targeting either the lateral (H1 coil) or medial (H7 coil) PFC. The intent-to-treat (ITT) sample included 169 patients, and the completers (CO) sample included 143 patients (Figure 1B). A total of 19 patients who initiated treatment (6 randomized to H7 coil and 13 to H1 coil) did not complete the study, and the time to early termination was shorter in the H1 coil group than in the H7 coil group (log-rank test *P* value = 0.0045). The main reasons for early termination are presented in Supplemental Table 1.

The ITT sample included mostly White (80.5%) and female patients (60.9%) with a mean age of 45.4 (SD = 11.67), who experienced 9.1 (SD = 18.42) previous depressive episodes and failure of 3.0 (SD = 1.93) antidepressants in the current episode. No baseline differences in demographics, depression history, or treatment history were observed between the groups (Table 1 and Supplemental Tables 2–4), but the average number of education years happened to be higher in patients randomized to the H7 coil group than those randomized to the H1 coil group (Supplemental Table 2).

### Efficacy endpoints.

**Figure 2:**
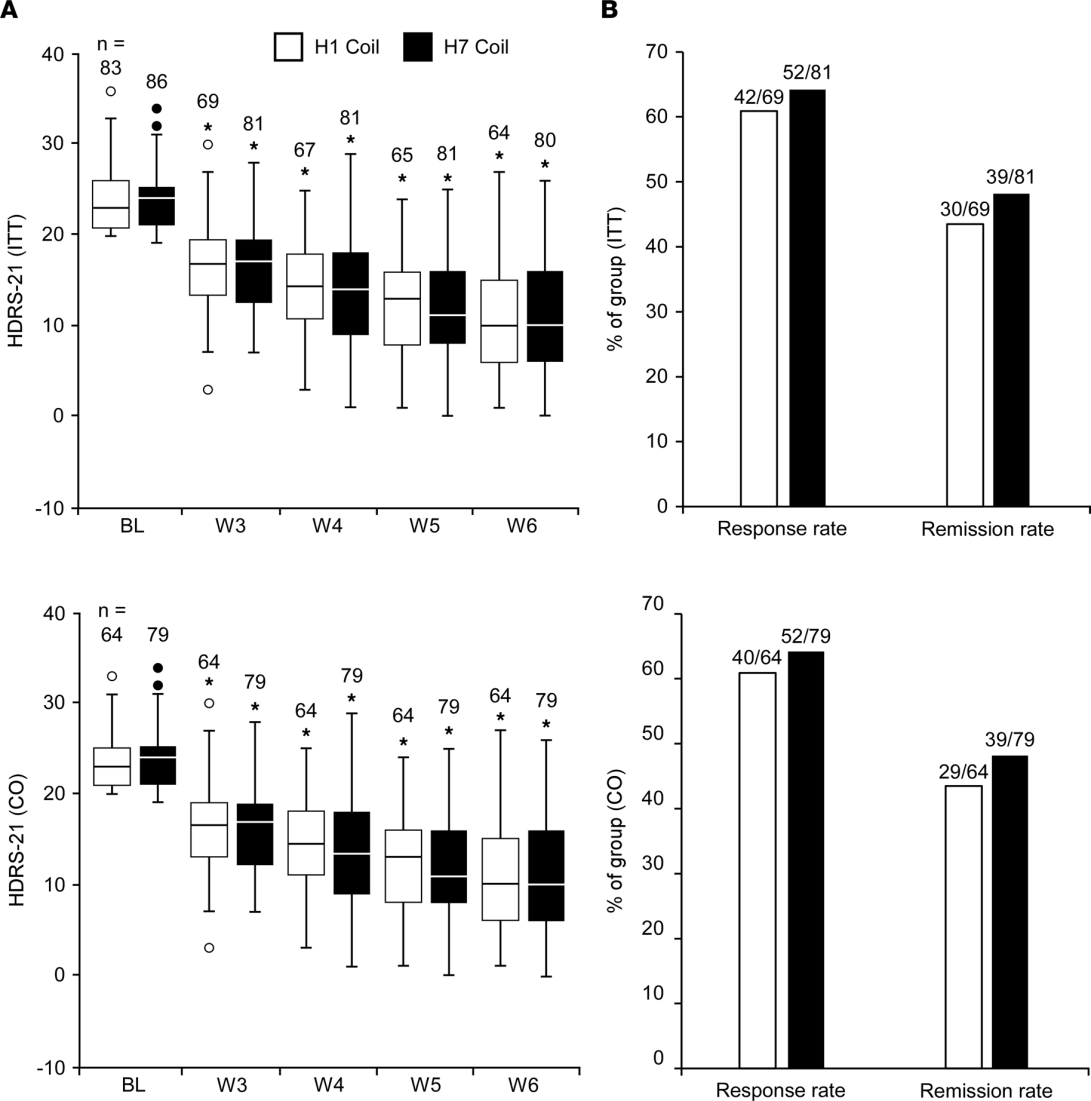
Response to treatment. Box-and-whisker plots for changes in HDRS-21 scores over the treatment course (**A**) and percentage of patients who experienced response (≥50% reduction from baseline in HDRS-21 score) and remission (HDRS-21 < 10) at the week 6 visit (**B**). Repeated measures ANCOVA models indicated that starting at the first measurement following treatment initiation (week 3), HDRS-21 scores were significantly reduced from baseline in both groups. No differences were observed between groups at any time point. The box plots depict the minimum value, first quartile, median, third quartile, and maximum value. The length of the box represents the interquartile range and dots represent outliers. *P < 0.0001, a repeated measures ANCOVA coefficient test for the change from baseline scores of that group. BL, baseline; W, week.

The primary efficacy endpoint of the study was the change from baseline to week 6 in the HDRS-21 scores. In both the ITT set and the CO set (Figure 2A), HDRS-21 scores gradually and significantly decreased from baseline to week 6, with scores at weeks 3–6 significantly lower than baseline scores in both treatment groups, but with no differences observed between the groups at any time point (Supplemental Tables 5–8). The difference between groups in the HDRS-21 change from baseline to week 6 was 0.229 and 0.026 points (for the ITT and CO sets, respectively), and the upper limit of the 1-sided 95% CI was lower than 3.0 HDRS points (i.e., 1.918 and 1.745 for the ITT and CO sets, respectively; Supplemental Table 9), validating that the efficacy of the H7 coil was noninferior to that of the H1 coil.

The secondary efficacy endpoints were response and remission rates at the week 6 final visit. In the ITT set, response rates were 60.9% following treatment with the H1 coil and 64.2% following treatment with the H7 coil. Remission rates were 43.5% following treatment with the H1 coil and 48.1% following treatment with the H7 coil. In the CO set, response rates were 62.5% and 65.8%, and remission rates were 45.3% and 49.4% for the H1 and H7 coils, respectively (Figure 2B). There were no significant differences between groups across analysis sets and H-coils.

Exploratory analysis of the change from baseline to week 6 in other clinical measures, including HARS, found significant within-group reductions, but no between-group differences, in both the ITT and the CO sets (Table 2).

### Safety.

No differences were found for safety parameters between the H-coils (Supplemental Tables 10 and 11), based on the YMRS, SSI, MMSE, and BSRT assessment scales. Overall, 125 participants reported adverse events at any time point during the study course, 60 (out of 83, 72.3%) in the H1 coil group and 65 (out of 86, 75.6%) in the H7 coil group, with no significant differences between groups. The most common complaints were headaches during the first treatment sessions and application site discomfort, in line with former TMS studies (7, 22).

### C-DEPTH.

Medial and lateral stimulation are expected to differently affect brain activity. Therefore, while both treatments were found to be similar in their overall effectiveness across all measures, the 2 stimulation sites may differentially treat distinct populations with MDD. To explore this, we analyzed the relation between baseline (pretreatment) symptom cluster severity and response to 1 H-coil (brain target) over the other. Informed by recent evidence of the H1 coil’s polysymptomatic effect on depression and anxiety symptoms (11, 37, 38), we used a baseline cluster consisting of 8 depression and anxiety HDRS-21 items (items 1, 7–11, 13, and 15) established in the literature through factor analysis (43), which underwent leave one feature out (LOFO) optimization to include 3 depression-related items (1, depressed mood; 7, work and interests; 8, retardation) and 3 anxiety-related items (9, agitation; 10, anxiety psychic; 11, anxiety somatic), as detailed in Supplemental Data 2, “Correlation between C-DEPTH and percent change in HDRS-21 score.”

**Figure 3:**
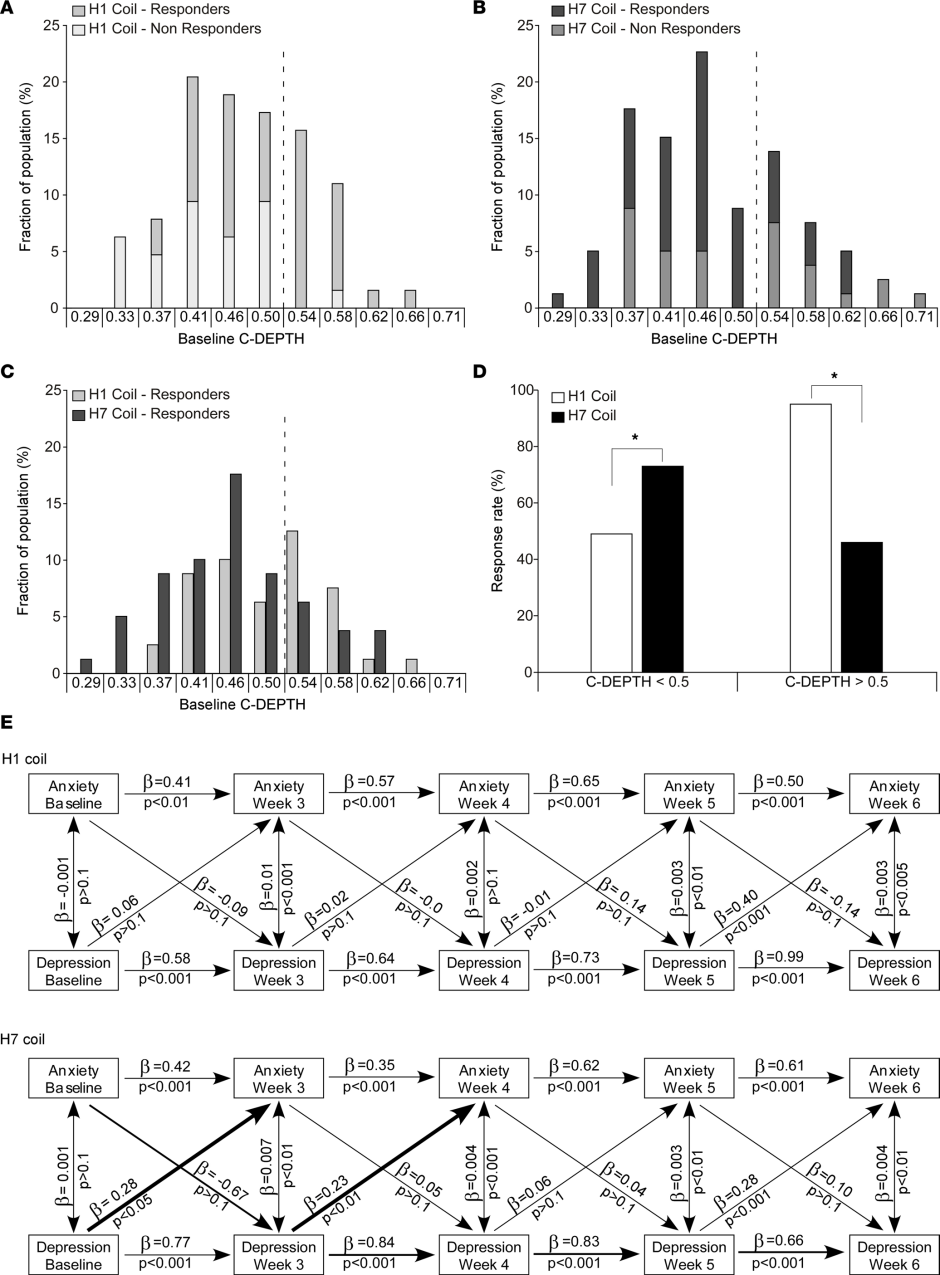
Baseline C-DEPTH and clinical response. Histograms of C**-**DEPTH for patients who received H1 coil stimulation (**A**) and H7 coil stimulation (**B**) grouped into responders and nonresponders. (**C**) Histogram of C**-**DEPTH scores for patients with clinical response. (**D**) Response rate in patients with C**-**DEPTH scores above or below the 0.5 cutoff value. *P < 0.05 following a χ^2^ test. Response rates significantly differ between treatments both below (χ^2^_1,__N__=__101_ = 6.3, *P* = 0.01) and above (χ^2^_1,__N__=__43_ = 11.5, *P* = 0.0068). (**E**) Cross-lagged regression analysis for the anxiety and depression subscales of the C**-**DEPTH over the course of the treatment (*n* = 144). The cross-lagged regression analysis was performed in R, version 3.6.1, using the lavaan package. Wide arrows represent statistically significant cross-lagged waves that differ between groups.

Baseline C-DEPTH scores were significantly and positively correlated with treatment-induced reduction in HDRS-21 scores following treatment with the H1 coil (*r* = 0.37, *P* = 0.003) but negatively and nonsignificantly with the H7 coil (*r* = –0.09, *P* = 0.45; Supplemental Figure 1). The different relations between baseline C-DEPTH and clinical response for the different H-coils suggests that the H1 coil may be more effective for patients with higher C-DEPTH scores. An optimal threshold of C-DEPTH = 0.5 results in an odds ratio of 21 in favor of the H1 coil for C-DEPTH > 0.5 (*n* = 43; *P* = 0.006) and an odds ratio of 2.8 in favor of the H7 coil for C-DEPTH ≤ 0.5 (*n* = 101; *P* = 0.01; Figure 3, A–C). In patients with C-DEPTH > 0.5, this translates to response rate of 95% for those treated with the H1 coil and only 46% for those treated with the H7 coil (χ^2^_1,__N__=__43_ = 11.5, *P* = 0.0068). On the other hand, in patients with C-DEPTH ≤ 0.5, response rates are 73% for those treated with the H7 coil but only 49% for those treated with the H1 coil (χ^2^_1,__N__=__101_ = 6.3, *P* = 0.01; Figure 3D).

By splitting the predictive cluster into depression and anxiety, we observed that this prediction was primarily driven by the anxiety subcluster for the H1 coil and tended to be driven by the depression subcluster for the H7 coil (see Supplemental Data 2, “Correlation between C-DEPTH and percent change in HDRS-21 score”). As such, we also explored whether the H-coils interact differently with the depression and anxiety subclusters of the C-DEPTH over the course of the treatment. A cross-lagged regression analysis found that during the first 4 weeks of daily treatment, there were significant crossed-lagged waves from previous measures of depression to measures of anxiety in patients treated with the H7 coil, but not with the H1 coil, suggesting a different time course and potentially a different mechanism of action for the medial and lateral stimulation (Figure 3E).

While the C-DEPTH threshold was constructed for the prediction of response rates for the different H-coils, it also revealed a trend for predicting remission rates. Patients with C-DEPTH > 0.5 had remission rates of 58% following treatment with the H1 coil and only 33% following treatment with the H7 coil (χ^2^_1,__N__=__43_ = 2.6, *P* = 0.1), whereas patients with C-DEPTH ≤ 0.5 had remission rates of 40% following treatment with the H1 coil but 55% following treatment with the H7 coil (χ^2^_1,__N = 101_ = 2.4, *P* = 0.12).

In an attempt to validate C-DEPTH ability to predict response to the H1 coil (no corresponding database is available for the H7 coil, which makes a true validation impossible), we tested outcomes of an independent data set collected under similar conditions (44). The study included 72 patients with MDD who completed 4 weeks of treatment with the H1 coil and reported a 67% response rate with the HDRS-17. In accordance with predictions, patients in the ITT set with C-DEPTH > 0.5 (*n* = 18) had a higher-than-population-average response rate (83%), while those with C-DEPTH ≤ 0.5 (*n* = 54) had a lower-than-population-average response rate (61%: χ^2^_1,__N = 72_ = 3, *P* = 0.08). In the per protocol (PP) set of that study, patients with C-DEPTH > 0.5 (*n*= 16) had a higher-than-average response rate (94%) while those with C-DEPTH ≤ 0.5 (*n* = 49) had a lower-than-average response rate (65%). The difference in response rates between the 2 side of the threshold was significant (χ^2^_1,__N = 65_ = 4.9, *P* = 0.03). Remission rates in the ITT set were 72% for patients with C-DEPTH > 0.5 and only 55% for patients with C-DEPTH ≤ 0.5 (χ^2^_1,__N = 72_ = 1.6, *P* = 0.21) while in the PP set remission rates were 81% and 59%, respectively (χ^2^_1,__N = 65_ = 2.6, *P* = 0.11).

### E-DEPTH.

**Figure 4:**
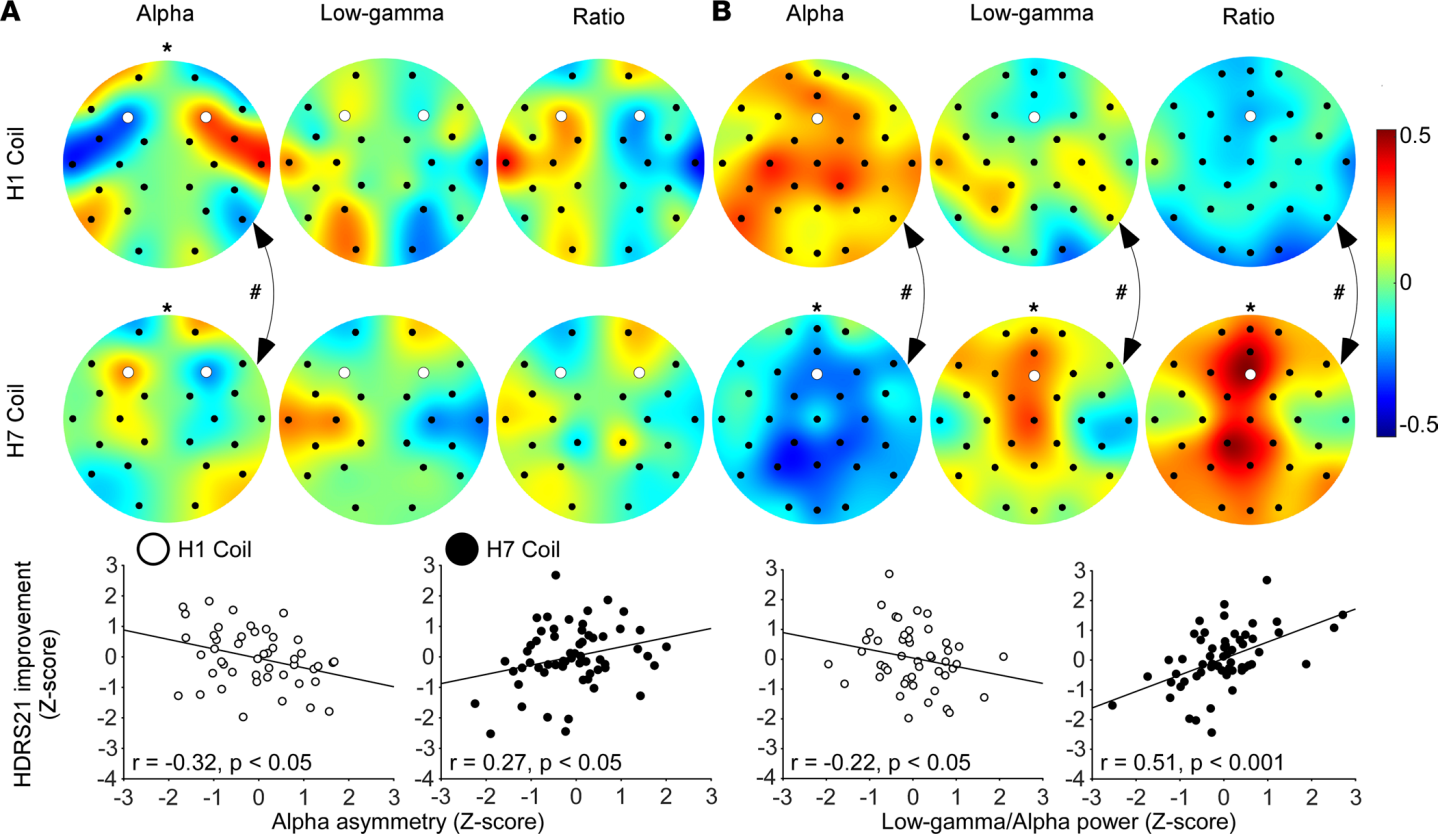
Correlation between clinical improvement and brain activity during the first treatment. (**A**) Topographical plots of the correlation between improvement in HDRS-21 score and brain asymmetry in the alpha band, low-gamma band, and low-gamma/alpha ratio and scatterplots of left alpha band asymmetry over the LPFC (electrodes F3 and F4, marked white. H1: *n* = 48; H7: *n* = 59). (**B**) Topographical plots of the correlation between absolute brain activity and improvement in HDRS-21 score and scatterplots of the power ratio over the MPFC (electrode Fz; marked white. H1: *n* = 48; H7: *n* = 58). Panel arrangement is similar to **A**. * represents significant linear correlation test, and ^#^ represents significant Fisher’s *Z* test for differences in correlation magnitude between H1 and H7 coils.

Following recent work indicating that alpha and low-gamma EEG bands during TMS treatment may represent the responsivity of the cortex to TMS (and predicted response to Deep TMS in ADHD patients, ref. 42), we analyzed the power of these EEG bands during the first treatment session and examined whether they can also constitute an E-DEPTH in MDD patients (also see Supplemental Methods, “EEG recording and preprocessing methods”). In accordance with our a priori hypotheses, the clinical effects of the H1 coil were correlated with lateralized PFC asymmetry during the TMS session, while those of the H7 coil were correlated with medial absolute PFC activity. However, the clinical effect of the H7 coil was also unexpectedly correlated with asymmetric activity. More specifically, increased left asymmetry of the alpha band was negatively correlated with the clinical improvement induced by the H1 coil (*r* = –0.32, *P* = 0.03) but positively correlated with the clinical improvement induced by the H7 coil (*r* = 0.27, *P* = 0.04). The difference between these opposite correlations in the H1 and H7 coil groups was statistically significant (*Z*_Fisher_ = 3.02, *P* = 0.002; Figure 4A). In addition, absolute activity power of the alpha band, low-gamma band, and low-gamma/alpha ratio over the MPFC-ACC were significantly correlated with the clinical improvement induced by the H7 coil (*r* = –0.29, *P* = 0.03; *r* = 0.28, *P* = 0.03; and *r* = 0.51, *P* = 0.0003, respectively); but none of these were strongly correlated with the clinical improvement the H1 coil induced, and the differences in magnitude of correlations between the 2 H-coil groups were significant (*Z*_Fisher_ = 2.61, *P* = 0.009; *Z*_Fisher_ = 2.08, *P* = 0.03; *Z*_Fisher_ = 3.89, *P* = 0.0001, respectively; Figure 4B).

Treatment current source density (CSD) data verified that the correlation of absolute activity power over the MPFC-ACC to the clinical outcome following stimulation with the H7 coil was caused by local activity, yet no such verification was achieved for LPFC stimulation with the H1 coil (Supplemental Figure 2). Additionally, a negative correlation was found in the pretreatment resting state activity low-gamma/alpha power ratio of the H1 coil but not the H7 coil groups, mainly due to positive correlation of the alpha band component (Table 3 and Supplemental Figures 3 and 4).

## Discussion

The main clinical finding of the present multicenter study is that treatment of MPFC-ACC with the H7 coil in patients with MDD, who did not respond to antidepressant medications in the current episode, produced a strong and noninferior effect relative to that induced by LPFC stimulation with the H1 coil. Both H-coils induced similar decrease of depression and anxiety levels, similar increase in quality of life, and similarly high response and remission rates in these patients with MDD. As expected from a study including 2 active arms, response and remission rates were higher than those achieved in the original sham-controlled study that led to the clearance of the H1 coil by the FDA (7) and were similar to those of postmarketing assessments for the efficacy of the H1 coil in patients with depression (45). As such, the results obtained here may approximate those expected in real-world clinical practice.

We also identified an easy-to-use C-DEPTH for the clinical outcome, composed of mood and anxiety items derived from HDRS-21 (8, 27, 46, 47), whose baseline severity may serve for patient selection between the H-coils. Patients with a combination of high baseline depression and anxiety (C-DEPTH > 0.5) responded better to the H1 coil, an effect that is corroborated in an independent data set (44). This is consistent with a recent analysis of the H1 coil multicenter RCT for MDD that shows baseline anxiety to be a positive predictor of effect on depression and anxiety (48) and with a prospective head-to-head study, where response rate induced by the H1 coil is significantly higher than that induced by a traditional figure eight coil in patients with moderate to severe depression (44). Interestingly, high scores of depression (49–51) have been shown to be a negative predictor for treatment of MDD in the FDA-cleared protocols using the figure eight coil (using either high frequency or theta burst stimulation) (32), and high baseline anxiety was shown to be a negative predictor of efficacy for both traditional figure eight TMS (52, 53) and pharmacotherapy (53). In accord with that, patients with a combination of low baseline depression and anxiety (C-DEPTH ≤ 0.5) responded better to the H7 coil, which is in line with evidence that abnormal functional connectivity in the MPFC is associated with a subtype of depression with manifestation of low anhedonia and anxiety (4).

The construction of an E-DEPTH model is nuanced, as data for each group include response to only 1 H-coil (converse to the uniform C-DEPTH for both groups). Nevertheless, individual outcome scores were correlated with activity in a coil-specific manner. As expected, alpha activity over the stimulated area of each coil, which was suggested to reflect cortical inhibition level (54), negatively correlated with the clinical outcome (Figure 4). This suggests that patients with lower levels of alpha asymmetry during the first H1 coil session, or lower medial alpha power during the first H7 coil session, are more likely to benefit from treatment. For the H7 coil only, patients with higher medial low-gamma power (suggested to depict the cortical response to the high-frequency stimulation; ref. 55), or higher medial low-gamma/alpha power ratio, were more likely to benefit from treatment, with the ratio demonstrating the highest correlation with the clinical outcome (Figure 4 and Supplemental Figure 2). The between-coil differences in correlation type (asymmetry versus power) suggest that LPFC stimulation interacts with the activity balance between the 2 hemispheres, while MPFC-ACC stimulation interacts with the sheer brain activity under the stimulated site (42). The former difference may occur due to interhemispheric tracts connecting between homological areas of the 2 hemispheres through the corpus callosum (56), which form an inhibitory relationship between the homologous areas, giving rise to phenomena like hemispherical dominance and interhemispheric inhibition (57).

Together, the electrophysiological correlates for response to Deep TMS in this study support and extend findings from a previous study in patients with ADHD treated by a different H-coil (42), which add to the validity of alpha and low-gamma activity during stimulation as predictors for clinical response to Deep TMS and suggest different mechanisms of action of the H-coils (see extended discussion of the electrophysiological data in Supplemental Data 2, “Supplementary EEG Discussion.” Indeed, while left lateralization of the alpha band negatively correlated with response to the H1 coil, it was positively correlated with response to the H7 coil. These effects may be due to the different interactions of the 2 H-coils with the LPFC nodes of the central executive network (CEN) on the one hand (58) (H1 and H7 coils) and the MPFC node of the default mode network (DMN) on the other (59) (H7 coil only). The CEN and DMN networks are anticorrelated during resting state (60, 61), and excitation or inhibition of the CEN using TMS causally inhibits or disinhibits the DMN, respectively (62).

This may be in line with results of the cross-lagged regression model, which revealed that depression and anxiety were treated independently and simultaneously by the H1 coil, while the H7 coil demonstrated a different and distinct pattern of a lagged reduction of anxiety that followed a decrease in depression. Together, the deeper and broader effective electric fields of Deep TMS relative to traditional figure eight coils, and the different interactions of the H1 and H7 coils with brain activity and symptoms’ alleviation, may provide an explanation for the advantage of the H1 coil in patients with more severe C-DEPTH, compared with the H7 coil, figure eight coil, and pharmacotherapy.

It is interesting to compare the efficacy of the H7 coil targeting the MPFC-ACC with previous TMS studies in MDD patients that targeted these brain areas. In previous open studies or RCTs that did not include a sham arm (16–19), which included overall 482 MDD patients treated with the D-B80 coil over the MPFC, the weighted average response and remission rates were 41.4% and 31.5%, respectively, which are lower than the rates of 64.2% and 48.2% obtained in the present study following treatment with the H7 coil. Comparison outcomes in various trials are limited because of variations in the protocol such as number of pulses, stimulation frequency, and precise positioning and orientation, yet this discrepancy may be at least partially attributed to the much deeper and broader stimulation of the H7 coil, which recruits many more MPFC-ACC structures associated with the reward system (21).

A limitation of the C-DEPTH and E-DEPTH is a lack of crossover data in which patients were selected for treatment based on their baseline clinical score or brain response during a single session (23). As such, together with the limited sample size of the current study, these predictors should be treated as preliminary. It is likely that the C-DEPTH will be improved following additional collection of data in future studies, particularly with the H7 coil, such that a more established formula for allocation of patients could be made. Nevertheless, following the novel FDA clearance for the treatment for MDD using the H7 coil (resulting from the present study), such preliminary predictors may provide initial guidance for clinicians in their decision between application of lateral (H1) or medial (H7) stimulation, avoiding random decisions. Therefore, even if our findings are preliminary and at risk of false positives, C-DEPTH may provide added value that may transcend the limitations. A limitation of the E-DEPTH is the lack of practical cutoff values that can be derived and recommended based on the present sample size. Obviously, while additional data can also optimize and improve the E-DEPTH, a major limitation of E-DEPTH is that neuroimaging and electrophysiological techniques such as EEG are not yet readily accessible to most clinics (36), and thus a predictor based solely on a common depression questionnaire is likely to prove more valuable and practical for standard clinical practice.

Taken together, our main findings are that MPFC-ACC stimulation using the H7 coil is an effective tool for the treatment of patients with MDD who did not respond to pharmacological treatment in the current episode and that the effectiveness of this intervention is noninferior to that induced by the FDA-cleared LPFC stimulation using the H1 coil. However, patients expressing higher severity in a cluster combining depression and anxiety symptoms (C-DEPTH > 0.5) responded better to LPFC stimulation with the H1 coil, while those expressing lower severity of this cluster (C-DEPTH ≤ 0.5) responded better to MPFC-ACC stimulation with the H7 coil. The present study also indicates potentially different underlying mechanisms of action for lateral and medial stimulation. By identifying specific clinical and electrophysiological predictors for response to each intervention, this study serves as an important step toward personalized TMS treatments of MDD.

## Methods

### Study overview.

This study was a prospective, 6-week, double-blind, randomized, controlled, multicenter trial in outpatients recruited in both academic and private research centers. The trial protocol was approved by local institutional review boards and was registered on ClinicalTrials.gov (NCT03012724). The study was conducted in the United States (6 sites), Israel (1 site), Canada (1 site), and Croatia (1 site), with active enrollment and treatment from May 2017 through August 2021. The trial was supported by industry (BrainsWay Ltd.). Full methods and statistical description are available in the Supplemental Methods.

### Patients.

Patients were treated as outpatients with ages from 22 to 69 years who signed an informed consent form and had HDRS-21 ≥ 20 that was stable between screening and baseline assessments (±30%; Figure 1). Only patients who did not respond in the current episode to at least 1 and up to 4 antidepressant drug trials were eligible for this study (further details on inclusion and exclusion criteria are provided in the Supplemental Methods). Main exclusion criteria included comorbid psychiatric and neurological disorders, presence of psychosis, primary anxiety disorder causing higher distress than MDD, substance abuse within 6 months, prominent personality disorder causing higher distress than MDD, dysthymia, prior head trauma or seizures, and suicide attempt within 3 years. Mood stabilizers and antipsychotics were not allowed. Antidepressants were allowed but had to be maintained at a stable dose for at least 2 months before enrollment and throughout the study.

### Randomization and blinding.

Patients were stratified per center by severity of disease as determined by baseline HDRS-21 score and the antidepressant treatment history form (ATHF). Thereafter, patients who met the eligibility criteria were equally allocated (with a 1:1 ratio) to 1 of the 2 treatment groups, stratified by HDRS-21 scores (20–25 vs. ≥ 26), ATHF categories (ATHF ≥ 2 level 1–2 and ATHF 1 level 3 or higher vs. ATHF 2–4, level 3 or higher), and center, based on a random-number generator.

Patients were told that they would receive 1 of 2 active treatments that differ in their parameters. The operator administering the treatment was the only one of the study personnel who was aware of the assigned group by code (A vs. B, as detailed below), while other study personnel and patients were completely blinded to the treatment being administered.

### Deep TMS treatment.

After insertion of earplugs, the individual resting motor threshold (rMT) was determined by locating a “hot spot” on the motor cortex for stimulation of the right abductor pollicis brevis using single pulses at 0.2 Hz. For treatment the H-coil was advanced 6 cm anteriorly over the prefrontal cortex, with the H1 (labeled as “A”) remaining over the LPFC and the H7 coil (labeled as “B”) adjusted to a centralized position before treatment. Patients and operators were not aware of the differences between coil A and B. The rMT was rechecked at least once a week. Treatment intensity was 120% of rMT.

During 4 consecutive weeks (5 sessions/wk; Figure 1) patients were treated daily while during the 2 additional weeks patients were treated biweekly (with at least 48 hours between treatments). Patients in both treatment groups received a similar Deep TMS protocol (120% of rMT, 18 Hz, 2 seconds on and 20 seconds off over a 20-minute period; total of 1,980 stimuli per session), applied over the lateral (H1 coil) or medial (H7 coil) PFC. Maps of the electric field distribution generated in both treatment modes are provided in Supplemental Figure 5.

### Assessments.

The primary efficacy endpoint of the study was the change from baseline in HDRS-21 scores at the week 6 visit. The secondary efficacy endpoints were response rate (≥50% reduction from baseline in HDRS-21 score) and remission rate (HDRS-21 < 10) at the week 6 visit. For a full list of assessments please see Figure 1.

### C-DEPTH.

Analysis was performed in Python, version 3.8, on the completers within the ITT set. Symptom clustering is generally done through factor analysis or machine learning with results that vary depending on the technique as well as the specific data sets analyzed (26, 28). While there are advantages for physiologically based or hypothesis-driven approaches, factor analysis and machine learning methods usually require larger sample sizes than that of the current study (although being relatively large for an interventional medical device study, still too small for common machine learning methods). As such, while the clustering is performed in an unsupervised fashion, the ratification and naming of the clusters are dependent on the input and experience of the researchers (26).

Given the evidence of the H1 coil’s polysymptomatic effect of treating depression and anxiety symptoms simultaneously (11, 37) and the effectiveness of the H7 coil in OCD treatment (22, 40), we purposely chose baseline clusters from the literature that included both anxiety and depression items of the HDRS-21 (27, 43, 47). Specifically, we used the observed mood and anxiety cluster (HDRS items 1, 7–11, 13 and 15) from Uher et al. (43). The cluster was computed as the mean of the normalized item scores (i.e., dividing the score of every item by its maximum value, yielding a range of 0–1; see calculator at https://lifewp.bgu.ac.il/wp/azangen/wp-content/CDEPTHcalculator.html). We then performed a further stage of optimization using a LOFO algorithm to determine whether both depression and anxiety items were contributing to the predictions, which resulted in removing items 13 and 15 from the calculated score. In addition, we calculated the linear regression of the optimal cluster with percentage change in HDRS score from the baseline to the study endpoint.

To validate the predictor, we tested it on a replication data set of H1 coil data only, as there is currently no data set of H7 coil for the treatment of MDD (44).

### Cross-lagged regression models.

The cross-lagged regression analysis was performed in R, version 3.6.1, using the lavaan package, version 0.6-9 (63). For analysis, the depression items (items 1, 7, and 8) and anxiety items (items 9–11) subclusters were used.

### Data and materials availability.

All the individual participant data collected during the trial (after deidentification), study protocol, statistical analysis plan, informed consent form, clinical study report, and analytic code will be available upon request.

### Statistics.

The full statistical plan is presented in Supplemental Methods, “Statistical Analyses.” The change in HDRS-21 from baseline to the 6-week visit was compared between the treatment groups using a repeated measures analysis of covariance (ANCOVA, SAS MIXED procedure), with baseline HDRS-21 and ATHF category, center, and time used as covariates. The primary efficacy endpoint was assessed via a 1-sided 95% CI.

For sample size estimation, a noninferiority margin of 3 points (δ = 3) between the changes in HDRS-21 for the 2 treatment arms was chosen, as this is considered the threshold for a difference between treatments to be clinically meaningful (64).

Baseline demographics and clinical characteristics were compared between the study groups. For comparison of means (continuous variables), the 2-sample *t* test or the Wilcoxon rank-sum test were used, as appropriate. For comparison of proportions (categorical variables), the χ^2^ test or Fisher’s exact test was used, as appropriate. *P* < 0.05 was considered statistically significant.

Details regarding rationale, hypotheses, recording, preprocessing, and statistical methods for EEG are described in the Supplemental Methods. Briefly, EEG was acquired in the first day of treatment during resting state and treatment, with patients’ eyes closed. Processing was conducted using EEGlab version 14.1.2 (65) and designated scripts in Matlab 2020a release (MathWorks, Inc.). Treatment data sets of 1 specific site (18 sets) were entirely excluded due to hardware grounding failure, causing high levels of line noise during treatment. Other highly contaminated data sets were excluded from analysis, leaving a total of 127 resting state and 111 treatment files.

Two-second epochs, starting 1 second following trains’ ending (to avoid TMS-related artifacts induced by the stimulation), were extracted from the intertrain intervals of the treatment data. Additionally, 1-second epochs immediately adjacent to trains’ commencing were extracted for noise reduction purposes. Power spectral density was then computed using Welch’s method, and influence of transient noise was reduced by train-wise deviation of the posttrain activity power spectrum with that of pretrain activity. Spectral density was computed using 2 reference schemes: AVR and CSD used to investigate the possibility that prefrontal activity may originate from posterior sources (66).

Next, absolute activity power in the alpha (8–12 Hz) and low-gamma (30–40 Hz) bands was extracted, and asymmetric activity scores were calculated as ratio between activity power of each electrode and that of the homologous electrode in the counter hemisphere. Alpha, low-gamma, and low-gamma/alpha ratio of activity and asymmetry scores were then linearly correlated with improvement percentage in HDRS21 score following 6 weeks of treatment. Site was partialled out of the correlation to control for site-related variability due to electrophysiological noise (multiple amplifiers, EEG caps, EEG technicians) and possible differences in rating tendencies. Correlations were further stabilized, by removing observations deviating more than 3 SDs from the mean of residual scores or leveraging scores. Following a priori hypotheses, groups were tested for differences in correlation magnitudes using Fisher’s *Z* tests, in specific electrodes of interest (EOI), according to the maximal magnetic induction points of each H-coil: F3 electrode was defined as left prefrontal EOI of the H1 coil and electrode Fz as medial prefrontal EOI of the H7 coil.

### Study approval.

The trial protocol was approved by local institutional review boards of the participating study sitesand was registered on ClinicalTrials.gov (NCT03012724). Written informed consent was obtained from each participant in the study after the nature of the study and potential consequences of participation in the study were explained.

## Author contributions

AZ and MSG conceived the study. AZ, SZ, NBY, UA, AS, FD, YR, and MSG developed methodology. AZ, SZ, AT, NBY, UA, DMB, GG, HS, TG, TV, AB, IF, DF, AS, FD, YR, and MSG investigated. SZ, NBY, UA, and YR visualized data. AZ and AT acquired funding. AS, FD, and TG were administrators of the project. MSG, AZ, AS, and UA supervised. SZ, NBY, AZ, and UA wrote the original draft. AZ, SZ, AT, NBY, UA, DMB, GG, HS, TG, TV, AB, IF, DF, AS, FD, YR, and MSG reviewed and edited the draft.

## Supplementary Material

Supplemental data

ICMJE disclosure forms

## Figures and Tables

**Table 3:**
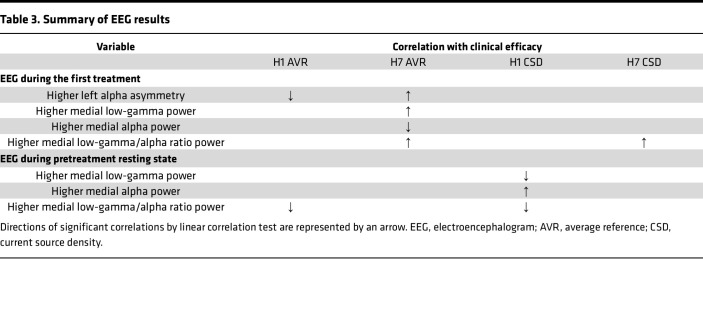
Summary of EEG results

**Table 2:**
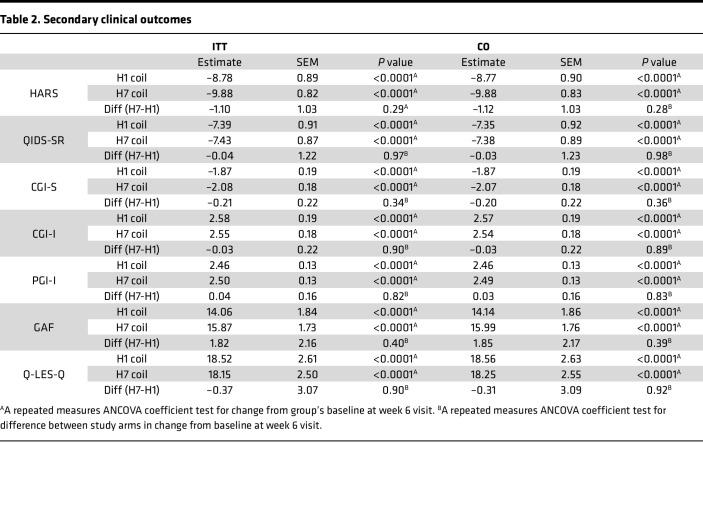
Secondary clinical outcomes

**Table 1:**
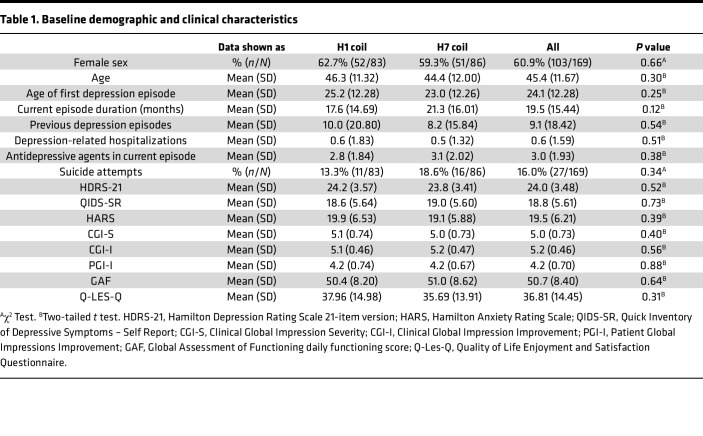
Baseline demographic and clinical characteristics

## Version 1 01/24/2023

In-Press Preview

## Version 2 02/22/2023

Electronic publication
