# Supplementary material for: Pursuing personalized medicine for depression by targeting the lateral or medial prefrontal cortex with Deep TMS
Source: JCI Insight. 2023 Feb 22;8(4):e165271. doi: 10.1172/jci.insight.165271 (PMC9977507; doi:10.1172/jci.insight.165271)
Supplement: Supplemental data [file jciinsight-8-165271-s210.pdf]

Supplementary Materials for

Pursuing personalized medicine for depression by targeting lateral or medial prefrontal cortex with Deep TMS

Abraham Zangen[*b], Samuel Zibman, Aron Tendler, Noam Barnea-Ygael, Uri Alyagon, Daniel M. Blumberger, Geoffrey Grammer, Hadar Shalev, Tatiana Gulevski, Tanya Vapnik, Alexander Bystritsky, Igor Filipčić, David Feifel, Ahava Stein, Frederic Deutsch, Yiftach Roth and Mark S. George[*a]

*Corresponding authors:

(a) Prof. Mark George, MD, Brain Stimulation Division, Psychiatry, Medical University of South Carolina, USA. Phone: 843 876 5142. Email: georgem@musc.edu

(b) Prof. Abraham Zangen, Ph.D. Department of Life Sciences and the Zlotowski Centre for Neuroscience, Ben-Gurion University of the Negev, Beer-Sheva 8410501, Israel. Phone: 972-8-6472646. Email: azangen@bgu.ac.il

1. Supplementary Introduction

1.1 EEG

Previous results of a medium sample size pilot study indicated that brain activity during the first treatment, correlates the level of therapeutic outcome following 3 weeks of daily TMS treatments directed unilaterally to the right pre-frontal cortex in ADHD patients (1). This predictive biomarker, computed as the power-ratio between low-gamma and alpha activity, formed a frontal inter-hemispheric asymmetrical pattern with symptomatic improvement, probing the activity balance between the two hemispheres, rather than absolute power. Further investigation of the biomarker dynamics during the inter-train interval, and its relation to resting-state EEG suggested that the low-gamma component reflects cortical response readily generated by TMS (2) while the alpha component indexes local cortical inhibition level (3). Together they mirror the responsivity of the cortex to TMS, and importantly are not activity components related to the ADHD pathology. Nevertheless, the pathology-independence of the biomarker was never investigated.

Furthermore, the low-gamma component of the biomarker may be alternatively explained by residual TMS related muscles activation (4) and distinction between these two alternatives (neural vs muscle originated) is hard to make. Thus, investigation of the generality of the biomarker in a large sample study – its existence in other psychiatric pathologies treated using different TMS coils directed to other brain areas - will provide strong converging evidence to support its validity.

Following the ADHD pilot results, study hypotheses were as follow: (1) Alpha and low-gamma activity during the first treatment will form a biomarker correlated with the therapeutic outcome following 6 weeks of daily Deep TMS treatment in depression patients. Alpha activity

will be negatively, while low-gamma activity and low-gamma/alpha power ratio positively, associated with therapeutic improvement. (2) The biomarker will reflect the different H-Coil structures and stimulation areas. Specifically: (2a) H7 Coil targeting medial pre-frontal cortex will correlate with the absolute activity power of electrodes above this area, whereas (2b) the H1 Coil targeting dominantly the left pre-frontal cortex (similarly to H6 Coil targeting right pre-frontal cortex in the ADHD study) will correlate with the activity balance (asymmetry) in pre-frontal lateral electrodes.

2. Supplementary clinical results

2.1 C-DEPTH predictor

2.1.1 Correlation between C-DEPTH and percent change in HDRS-21 score

For the 8-items mood and anxiety cluster, an optimal threshold of 0.5 results in an odds ratio of 2 in favor of the H7 Coil below the threshold (p=0.08) and an odds ratio of 4.4 in favor of the H1 Coil above the threshold (p=0.045). After LOFO optimization removed two features (items 13 and 15), the resulting cluster with an optimal threshold of 0.5 results in an odds ratio of 2.8 in favor of the H7 Coil below the threshold (p=0.01) and an odds ratio of 21 in favor of the H1 Coil above the threshold (p=0.006). We calculated the Pearson's correlation coefficient between C-DEPTH and percent change in HDRS-21 score from baseline to week 6. We found that for participants treated with the H1 Coil there was a significant positive correlation (r = 0.37, p = 0.003), while for participants treated with the H7 Coil there was a non-significant negative correlation (r = -0.09, p = 0.45).

Since the cluster used in the predictor consists of both depression and anxiety items, we divided the cluster into depression (depressed, work and activities, retardation) and anxiety (agitation, anxiety-psychic, anxiety-somatic) (5-7) and explored the relative importance of each subcluster at baseline in the performance of the treatments.

For the anxiety C-DEPTH subcluster there was a significant positive correlation for participants treated with the H1 Coil ($r = 0.48$, $p < 0.001$) and a non-significant correlation for those treated with the H7 Coil ($r = 0.014$, $p = 0.90$). For the depression C-DEPTH subcluster there were non-significant negative correlations for those treated with the H1 Coil ($r = -0.015$, $p = 0.90$) and the H7 Coil ($r = -0.19$, $p = 0.085$). Taken together, this suggests that participants with C-DEPTH > 0.5 respond better with the H1 Coil primarily due to high baseline anxiety, while participants with C-DEPTH ≤ 0.5 respond better with the H7 Coil primarily due to low baseline depression.

2.1.2 Supplementary clinical discussion

The positive correlation of HDRS-21 percent change to the anxiety but not depression subcluster for the H1 Coil is unique compared to results of not only the H7 Coil but also traditional TMS with the figure-8 coil (8, 9) as well as pharmacotherapy (9) for which high baseline anxiety was shown to be a negative predictor of efficacy. The lack of negative correlation of HDRS-21 percent change with depression is likewise distinctive. The efficacy of the H1 Coil does not drop off with increased baseline depression but on the other hand also does not treat patients with low baseline depression any better as is observed with the H7 Coil.

2.2 Supplementary electrophysiological results

No correlation was found between C-DEPTH and left alpha prefrontal cortex (PFC) asymmetry (electrodes F3-F4) during H1 Coil treatment (r=0.13, p=0.39) or between C-DEPTH and the medial PFC absolute activity (electrode Fz) during H7 Coil treatment (r=0.11, p=0.39).

2.3 Supplementary EEG discussion

Within-treatment alpha activity under the medial PFC stimulation area of the H7 Coil was negatively correlated, while low-gamma activity positively correlated, with the therapeutic improvement following 6 weeks of treatment, forming together a low-gamma/alpha ratio which may explain the variability in treatment outcome. In a similar manner, alpha asymmetry under the left LPFC stimulation area of the H1 Coil negatively correlated with the therapeutic improvement. Although no correlation was observed in the low-gamma band asymmetry, these results are largely consistent with our a-priori hypotheses regarding the nature of brain activity during TMS treatment: The alpha component is suggested to reflect cortical inhibition level (3) while the low-gamma component depicts the cortical response to the high frequency magnetic stimulation (2). These results replicate and extend a previous pilot study (1) using a sample of ADHD patients that were treated with a different H-Coil design, and thus contribute to the converging evidence supporting the biomarker validity.

Furthermore, the between-coil difference in biomarker type (power versus asymmetry) suggests that medial PFC stimulation interacts with the sheer brain activity under the stimulated site, while lateral PFC stimulation interacts with the activity balance between the two hemispheres. In a similar manner, right PFC stimulation was previously found to interact with within-treatment frontal asymmetric activity in ADHD patients (1). This difference may occur

due to inter-hemispheric tracts connecting between homological areas of the two hemispheres through the corpus callosum (10), that form inhibitory relationship between the homologous areas, giving rise to phenomena like hemispherical dominance and inter-hemispheric inhibition (11).

CSD analysis confirmed that the H7 biomarker is caused by local brain activity and not spuriously reflects distant (posterior) generators. Yet, no such confirmation was achieved for the H1 asymmetric biomarker (Figure S2). Nevertheless, given that no strong posterior correlation was spotted in the first place, the innate limitation of any reference scheme including CSD (12), and the fact that correlation magnitudes between brain activity and treatment outcome evaluated using CSD were systematically inferior to those evaluated using average referenced pre-treatment resting state in two independent studies (the current and the ADHD study (1)), suggest that the latter is more appropriate for appraisal of within Deep TMS treatment related biomarkers.

Quite surprisingly, the correlations found under the stimulated areas of both H-Coils were accompanied by opposite patterns under the non-stimulated areas (i.e., a significant positive correlation with alpha asymmetry found in the lateral electrodes of the H7 group, and a negative trend with the absolute power in the medial electrode found in the H1 group). This observation could be the consequence of interconnected, yet competitive distributed networks, with known nodes under the frontal stimulation areas of the H-Coils, implicated in depression and in the treatment of depression using TMS (13-15): The Default Mode Network (DMN) with its medial pre-frontal node (16) on the one hand, and the Central Executive Network (CEN) with its dorso-lateral pre-frontal nodes on the other hand (17). Studies have suggested that the CEN negatively regulates the DMN: those networks are anticorrelated during resting state (18, 19), and excitation

or inhibition of the CEN using TMS causally inhibit and disinhibit the DMN respectively (20). Thus, a biomarker observed in one network may be negatively reflected in the other one. Yet, it is not clear whether this reflection is caused by the stimulation itself or alternatively by the spontaneous negative association between the two networks. The biomarker observed in the low-gamma/alpha ratio activity during resting state (Figures S3 and S4) suggests that at least in the H1 group the latter option prevails.

To conclude, this large sample study provides converging evidence supporting the validity of within-treatment alpha and low-gamma activity as a biomarker for TMS therapeutic outcome in multiple psychiatric pathologies. The specific brain activity constituting the biomarker (absolute power or asymmetry) depends on stimulation target, suggesting different mechanisms of stimulation action.

3 Supplementary methods

3.1 Inclusion Criteria

- Outpatients.
- Men and women 22-68 years of age.
- Primary DSM-V diagnosis of Major Depression, single or recurrent episode confirmed by the Structured Clinical Interview for the DSM-V (SCID-V), with the additional requirements of a current episode ≥4 weeks and CGI-S ≥4.
- Current depressive episode is less than 5 years in duration (the definition of an episode is demarcated by a period of ≥2 months in which the patient did not meet full criteria for the DSM-V definition of major depressive episode);
- Total HDRS-21 ≥20 and Item 1 score ≥2 at the screening visit;

- The patient did not respond to at least one antidepressant treatment, i.e., a minimum of 1 and a maximum of 4 antidepressant drug trials of adequate dose and duration (defined as a minimum level of 3 on the antidepressant treatment history form (ATHF)) in the current episode. Current episode for ATHF level 3 patients should be > 12 weeks.; or
  Patients who have not completed antidepressant trials of adequate dose and duration (defined as a level of 1-2 on the ATHF) due to intolerance to therapy if they have demonstrated intolerance to 2 or more antidepressant medications in the current episode.
- Capable and willing to provide informed consent
- Able to adhere to the treatment schedule.
- Patient is stable on medication for 2 month and is not expected to change medication during all study period
- Satisfactory safety screening questionnaire for transcranial magnetic stimulation.

3.2 Exclusion Criteria

- Investigators, site personnel directly affiliated with this study, and their immediate families (immediate family is defined as a spouse, parent, child or sibling, whether by birth or legal adoption);
- Individuals diagnosed by the Investigator with the following conditions (current unless otherwise stated):
  - Depression secondary to a general medical condition, or substance-induced;
  - History of substance abuse or dependence within the past 6 month (except nicotine and caffeine). Note that use of cannabis for medical reasons in a stable regimen is permitted as long as the investigator excludes abuse of the substance.

    - Any psychotic disorder (lifetime), including schizoaffective disorder, or major depression with psychotic features, bipolar disorder, eating disorder.
    - Post-traumatic stress disorder or obsessive-compulsive disorder (current or within the past year)
    - Current (within 12 months of baseline) generalized anxiety disorder, panic disorder or social anxiety disorder as assessed by the investigator to be primary, causing a higher degree of distress or impairment than MDD.
    - Presence (within 12 months of baseline) of a personality disorder (such as antisocial, schizotypal, histrionic, borderline, narcissistic) as assessed by the investigator to be primary, causing a higher degree of distress or impairment than MDD.
- Previous Deep TMS treatment failure
- Previous ECT treatment failure
- Patients suffering from hypo or hyperthyroidism based on a pre-study TSH test or confirmation via medical history and who are not medically stabilized.
- Individuals with a significant neurological disorder or insult including, but not limited to:
  - Any condition likely to be associated with increased intracranial pressure
  - Space occupying brain lesion
  - Any history of seizure EXCEPT those therapeutically induced by ECT
  - History of cerebrovascular accident
  - Transient ischemic attack within two years
  - Cerebral aneurysm or any other significant neurological disorder or insult
  - Dementia

    - Mini Mental State Exam score of less than or equal to 24
    - Parkinson’s disease
    - Huntington’s chorea
    - Multiple sclerosis
- Increased risk of seizure for any reason, including prior diagnosis of increased intracranial pressure (such as after large infarctions or trauma), or history of significant head trauma with loss of consciousness for greater than or equal to 5 minutes, or familial or personal history of epilepsy
- Individuals with hearing loss
- A positive and unmitigated response to any question on the Transcranial Magnetic Stimulation Adult Safety Screen questionnaire
- ECT treatment within 3 months prior to the screening visit
- History of treatment with Vagus Nerve Stimulation (VNS)
- History of treatment with Deep Brain Stimulation (DBS)
- Use of any investigational drug within 4 weeks of the randomization visit
- Use of any medication(s) listed on the Excluded Medication List within 2 weeks of the randomization visit
- Present suicidal risk as assessed by the investigator or significant suicide risk based on HDRS-21 item 3 score of 3 or 4, or significant suicide risk as assessed using the Scale for Suicide Ideation, or a history of attempted suicide in the last 3 years
- Any self-inflicted harm in the past 3 months not in the context of suicidal ideation
- Cardiac pacemakers, implanted medication pumps, intracardiac lines, or acute, unstable cardiac disease

- Intracranial implant (e.g., aneurysm clips, shunts, stimulators, cochlear implants, or electrodes) or any other metal object within or near the head, excluding the mouth, that cannot be safely removed
- Implanted neurostimulators
- History of abnormal MRI
- Known or suspected pregnancy
- If participating in psychotherapy, must have been in stable treatment for at least 3 months prior to entry into the study, with no anticipation of change in the frequency of therapeutic sessions, or the therapeutic focus over the duration of the Deep TMS trial
- Clinically significant laboratory abnormality, in the opinion of the Investigator based on CBC and biochemistry
- Women who are breast-feeding
- Women of childbearing potential and not using a medically accepted form of contraception when engaging in sexual intercourse

3.3 Randomization and blinding

Participants randomized into the study were stratified per center by severity of diseases as determined by the baseline HDRS-21 score and the ATHF. Thereafter, participants that meet the eligibility criteria were equally allocated (with a 1:1 ratio) to one of the 2 treatment groups, stratified by HDRS-21 scores (20-25 vs. ≥26), ATHF categories (ATHF ≥2 level 1-2 and ATHF 1 level 3 vs. ATHF 2-4, level 3) and center, based on a stratified randomization scheme using the permuted block method using the SAS (version 9.4) random number generator. The block size were random and study personnel were blinded to the randomization block size.

Central randomization was implemented in conducting this study. Each participant's number and treatment code were assigned into the Interactive Web Response System (IWRS), which generated a unique participant number and treatment allocation. Participants were told that they will receive one of two active treatments that differ in their parameters. The operator administering the treatment was the only study personnel who had access to the IWRS and was aware of the assigned and administered treatment. All other study personnel, including the investigator, independent raters and study participants were blinded to the treatment being administered.

3.4 EEG recording and preprocessing methods

EEG was acquired using TMS-compatible 32 (8 sites) or 64-channel (one site) amplifiers (Tmsi Ltd.) and WaveGuard caps (ANT Ltd.) with POz as ground and Cz as a common reference. Impedance was kept below 10 kOhm and signal was digitized at 2048 Hz using a 24-bit AD converter.

Data from within the first treatment session was filtered (1-100 Hz bandpass, and 58-62 Hz or 48-52 Hz notch FIR filter, according to the line frequency in the specific site). Data was manually scanned and noisy channels (M=0.6, SD=1.3; out of 32 channels in majority of files, i.e., less than 2%) or epochs containing residual TMS related artifacts were excluded (M=8.3, SD=6.8; out of 55 post-train segments, i.e., 15%). Horizontal eye movements and eye blinks were removed using infomax ICA (M=1.7, SD=0.77 of components excluded).

Resting state EEG was recorded for 300 seconds (30 initial and 10 final seconds were truncated to prevent state transitional influence). The pre-processing of the resting state data was similar to that of the treatment, except that epochs containing excessive eye movement related

low frequency activity (2-4 Hz), or muscle related high frequency noise (20-40 Hz), were rejected automatically. This strategy is preferred over ICA components' deletion when data is abundant (21). Nevertheless, a subset of 24 files containing high levels of eye movements were cleaned using ICA.

3.5 Statistical Analyses

3.5.1 Power Analysis

Based on initial findings of remission rates in MDD or bi-polar patients who previously failed to respond to Deep TMS treatment using the H1 Coil (22), we hypothesized that the H7 Coil produces a reduction in depressive symptoms that are not inferior to the H1 Coil. Thus, for sample size estimation we assumed that the mean change from baseline in HDRS-21 in the H1 Coil arm will equal -6.3 (SD = 6.6), based on the CTP-0001-00 study, PP analysis set (23) and the study provided in support of the FDA-cleared BrainsWay Deep TMS device in 510(k) K122288, and in the H7 Coil arm -7.0 (SD = 7.3). Under these assumptions, as well as non-inferiority margin of $\delta = 3$ (a difference of less than 3 points on the HDRS-21 scale is not considered clinically meaningful (24)), we initially calculated that a sample size of 45 subjects per treatment group (total 90) would provide 80% power to detect at 5% level of significance (one sided). Of note, this sample size also provides 96% power for the (two tailed) testing of correlations between the C-DEPTH and E-DEPTH and the clinical outcome, assuming a medium correlation magnitude of 0.5. According to our previous clinical experience, we estimated a drop-out rate of 15% and thus we determined a sample size of 106 randomized subjects.

An interim analysis was performed after 80 subjects completed the 6-weeks of the study. The Interim Analysis Committee (IAC) found, that based on the observed SD of the completed sample in sample size from 45 to 73 subjects per treatment group achieve the original targeted power of 80%, for the mITT and ITT estimates. As such the recruitment goal was modified to a total of 146 subjects. No safety issues were observed by the IAC.

3.5.2 Primary Efficacy Endpoint

The primary efficacy endpoint is the change from baseline to 6 weeks visit in HDRS-21 score. The change in HDRS-21 from baseline to 6 weeks visit is compared between the treatment groups using a repeated measures analysis of covariance (ANCOVA, SAS® MIXED procedure). Baseline HDRS-21, ATHF category at baseline, center and time are used as covariates. Baseline HDRS-21 scores are entered as continuous variables so that the potential for co-linearity problems will be minimized. Additionally, the center variable is grouped by country as US versus out of US (OUS) and the analyses as for center is repeated on this new variable.

The unstructured covariance matrix structure was used. If the model does not converge, then either the compound symmetry or autoregressive (whichever model has the lower AIC statistic) covariance matrix structure were used instead. At this time point (up to 6 weeks) we did not expect a high proportion of dropouts. Thus, any missing data at 6 weeks post baseline can be considered missing at random. Therefore, for this evaluation no imputation of missing data is considered beyond the model estimates. Nevertheless, should the missing at random assumption prove to be incorrect, a sensitivity analysis using one of the methods for data imputation mentioned was performed.

The principal statistical analysis was a comparison between the treatment groups, derived from the time by treatment interaction term from the model. The adjusted mean change from baseline in HDRS-21 scores at 6 weeks post randomization was estimated from the model (LS Means) for each group with respective two-sided 95% confidence limits. The difference between the adjusted means is presented together with one-sided 95% upper confidence limit.

Non-inferiority was assessed by comparing the non-inferiority margin of $\delta = 3$ to the upper limit of the one-sided 95% confidence interval for the difference between the reduction in HDRS of the two treatments (H7 Coil vs. H1 Coil).

3.5.3 Secondary Efficacy Endpoints

Response rate at the 6-week visit

Response is defined as a reduction in HDRS-21 score from baseline ≥50%.

The response rates at week 6 in the two groups is estimated and presented with unadjusted exact two-sided 95% confidence intervals.

Remission rate at 6-week visit

Remission is defined as HDRS-21 score < 10. The remission rates at week 6 in the two groups is estimated and presented with unadjusted exact two-sided 95% confidence intervals.

3.5.4 Handling of Missing Data

The study outcome is not evaluated for patients who drop out prior to randomization.

Patients who dropped out after one or more treatment, and have data available for the analysis (i.e., at least one post-baseline assessment) of continuous variables, were analyzed with a

repeated measures analysis of variance model using PROC mixed in SAS which can handle missing data at random. At the 6-week evaluation, we do not expect a high proportion of dropouts. Thus, any missing data at 6 weeks post baseline is considered missing at random. Additionally, imputation of data such as by Last Observation Carried Forward (LOCF) may harm linearity. Therefore, for this evaluation, no imputation of missing data is considered beyond the model estimates.

For categorical variables (such as response and remission rates at week 6) the LOCF concept was applied. Baseline characteristics of patients who drop out is evaluated by study group to evaluate the potential reason for differential drop out. Measures were taken to ensure that when a subject leaves the study, he/she will have an evaluation of the endpoints at that time.

Supplementary Figures

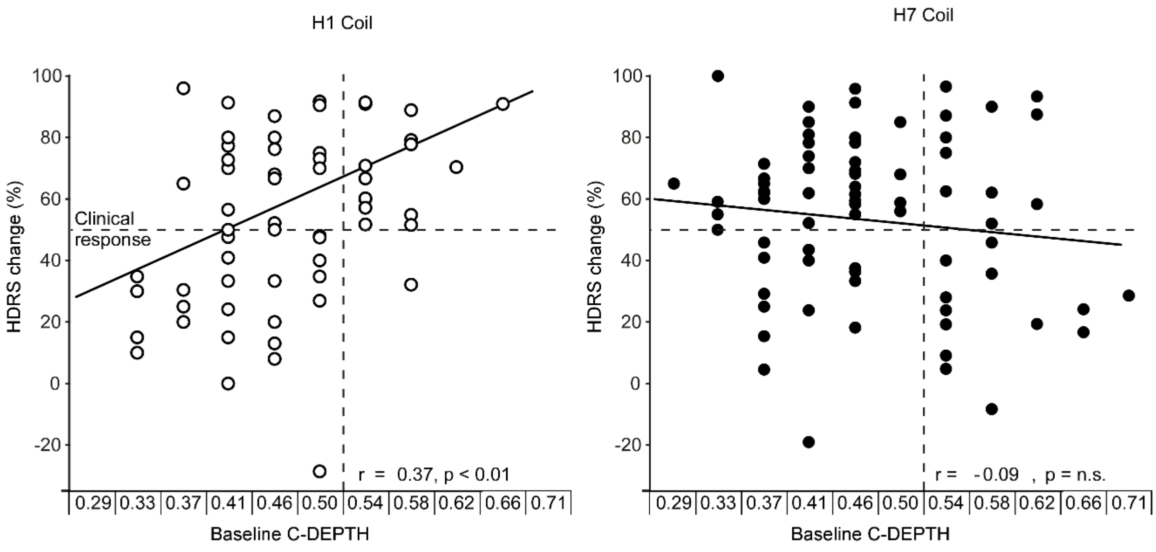

Figure S1. **Linear fit of percent HDRS change as a function of baseline mean normalized score**. For patients treated with the H1 Coil there was a significant positive correlation (Pearson's Correlation: r=0.37, p = 0.003, n = 64) while for patients treated with the H7 Coil, there was a trend for negative correlation (r = -0.09) although it was not significant (p = 0.45, n=80).

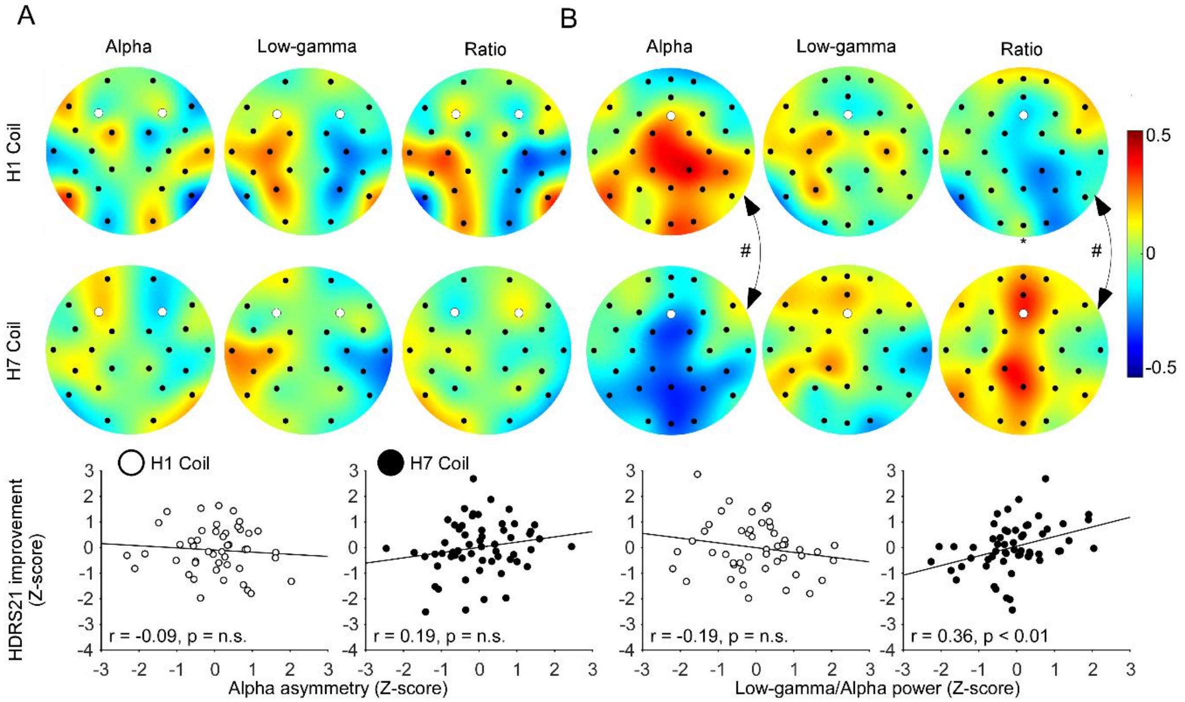

Figure S2. **Correlation between clinical improvement and brain Current Source Density (CSD) during the first treatment**. (a) Topographical plots of the correlation between improvement in HDRS-21 score and CSD asymmetry in the alpha band, low-gamma band, and low-gamma/alpha ratio; and scatter plots of left alpha band asymmetry over the lateral PFC (electrodes F3 and F4; marked white). (b) Topographical plots of the correlation between absolute CSD and improvement in HDRS-21 score; and scatter plots of the power ratio over the medial PFC (electrode Fz; marked white). Panel arrangement is similar to (a). * represent significant linear correlation test and # represent significant Fisher z test for differences in correlation magnitude between H1 and H7.

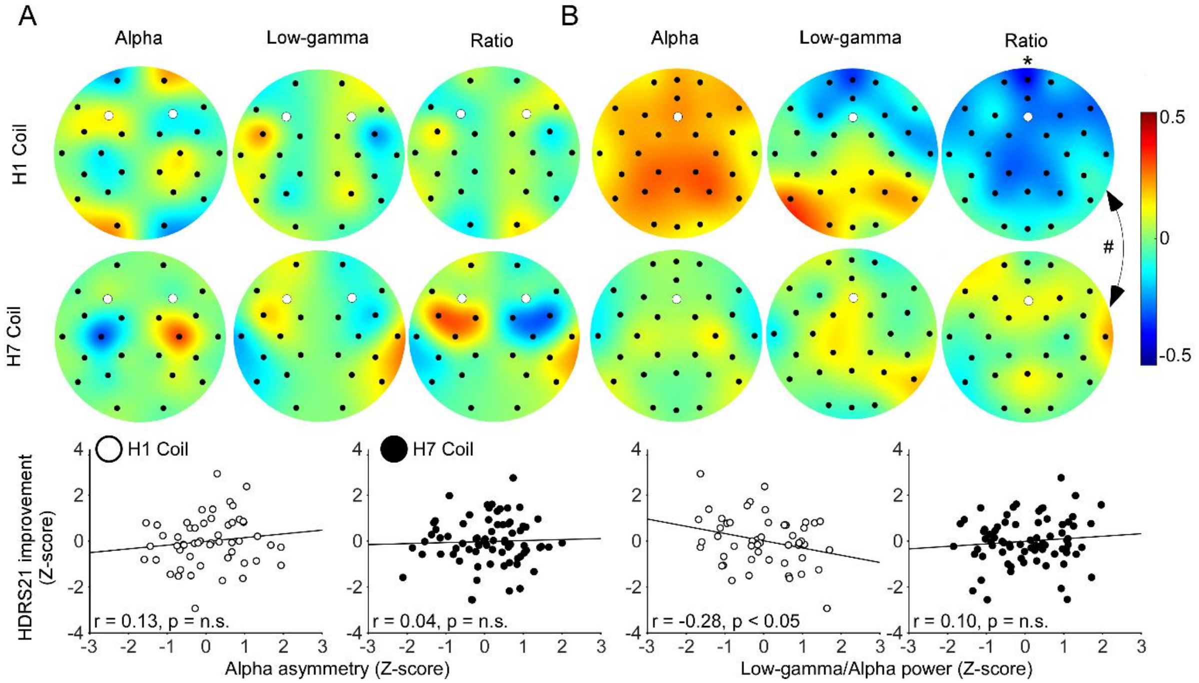

Figure S3: **Correlation between clinical improvement and average referenced (AVR) brain activity during the pre-treatment resting state.** (a) Topographical plots of the correlation between improvement in HDRS-21 score and resting state brain asymmetry in the alpha band, low-gamma band, and low-gamma/alpha ratio; and scatter plots of left alpha band asymmetry over the lateral PFC (electrodes F3 and F4; marked white). (b) Topographical plots of the correlation between absolute resting state brain activity and improvement in HDRS21 score; and scatter plots of the power ratio over the medial PFC (electrode Fz; marked white). Panel arrangement is similar to (a). * represent significant linear correlation test and # represent significant Fisher z test for differences in correlation magnitude between H1 and H7 Coils.

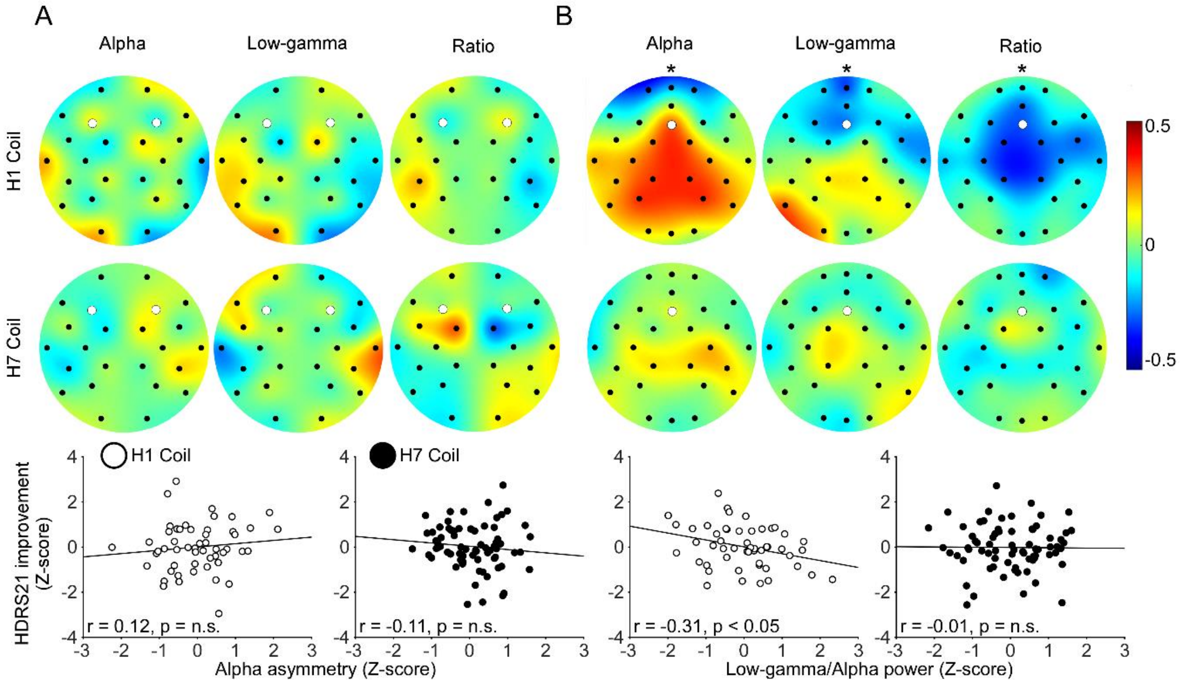

Figure S4: **Correlation between clinical improvement and brain Current Source density (CSD) during pre-treatment resting state.** (a) Topographical plots of the correlation between improvement in HDRS21 score and resting state CSD asymmetry in the alpha band, low-gamma band, and low-gamma/alpha ratio; and scatter plots of left alpha band asymmetry over the lateral PFC (electrodes F3 and F4; marked white). (b) Topographical plots of the correlation between absolute resting state CSD and improvement in HDRS-21 score; and scatter plots of the power ratio over the medial PFC (electrode Fz; marked white). Panel arrangement is similar to (a). * represent significant linear correlation.

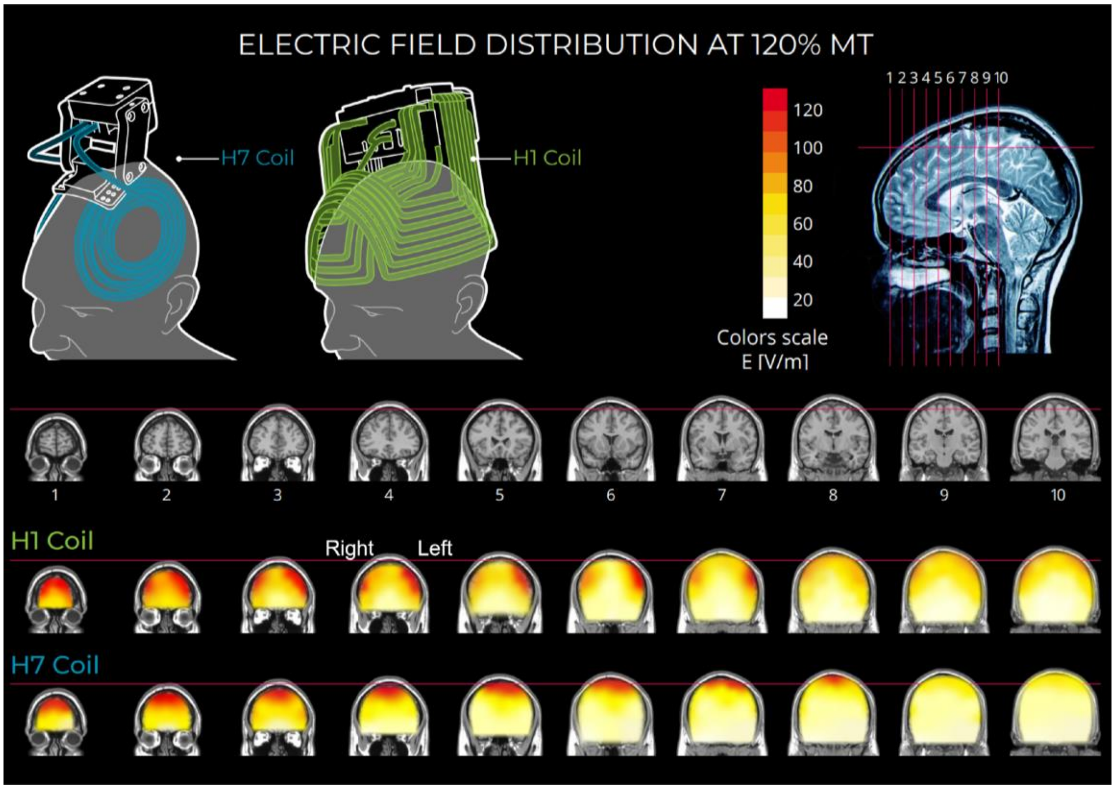

Figure S5. Distribution of the electric fields. Distribution of electric fields induced by the H1 and H7 Coils superimposed on coronal MRI slides . The electric field distribution was measured in a phantom model of the human head (15 x 13 x 18 cm), filled with physiologic saline solution. The colored field maps indicate the electrical field absolute magnitude in each pixel, for 10 coronal slices, 1 cm apart, along with the appropriate MRI coronal images. The H-Coils were placed over the theoretical frontal cortex of the head model and the field in each pixel was measured using a ‘pick-up’ dipole probe, attached to an oscilloscope. The red colors indicate field magnitude above the threshold for neuronal activation, which was set to 100 V/m based on the average threshold for motor activation of the hand. The field maps are adjusted for 120% of the motor threshold (120 V/m; as used in the current study), at the average depth of the hand motor cortex (1.5 cm), in accord with the protocol. When placed over the prefrontal cortex (as guided in the present study, applying intensity of 120% of the motor threshold), the H1 Coil induces suprathreshold fields (>100V/m, indicated by red pixels) in both left and right dorsolateral and ventrolateral prefrontal cortices, with greater distribution and penetration into the left hemisphere, while the H7 coil induces suprathreshold fields in the dorsomedial prefrontal cortex, as well as some of the ventromedial prefrontal and anterior cingulate cortices.

Supplementary Tables

Table S1. Main Reason for Early Termination (ITT)

	H1 Coil	H7 Coil	Total
**Inability to locate and quantify motor threshold (MT) as defined in the protocol.**	1	1	2
**Lost to follow-up**	.	2	2
**Withdrawal of consent**	9	3	12
**Subject is not compliant with requirements of the study including inclusion and exclusion criteria.**	2	.	2
**Subject developed documented suicidal ideation (based on HDRS-21 item 3 score is 3 or 4 or as assessed by the Suicide Ideation Scale) or attempts suicide after receiving H1 or H7 Deep TMS.**	1	.	1
**The investigator believes that for safety reasons (e.g., an adverse event) it is in the best interest of the subject to stop treatment.**	4	.	4
**The subject missed more than 3 treatments.**	1	.	1
**Other**	1	.	1

Table S2. Baseline demographic characteristics (ITT)

			H1 Coil	H7 Coil	All	p-value
**Sex**	**Male**	**% (n/N)**	37.3% (31/83)	40.7% (35/86)	39.1% (66/169)	0.6556 (#)
	**Female**	**% (n/N)**	62.7% (52/83)	59.3% (51/86)	60.9% (103/169)	
**Marital status**	**Married**	**% (n/n)**	51.8% (43/83)	34.9% (30/86)	43.2% (73/169)	0.0619 (#)
	**Single**	**% (n/N)**	26.5% (22/83)	45.3% (39/86)	36.1% (61/169)	
	**Divorced**	**% (n/N)**	19.3% (16/83)	16.3% (14/86)	17.8% (30/169)	
	**Widower**	**% (n/N)**	2.4% (2/83)	3.5% (3/86)	3.0% (5/169)	
**Race**	**Caucasian**	**% (n/N)**	80.7% (67/83)	80.2% (69/86)	80.5% (136/169)	0.9359 (#)
	**Afro-American**	**% (n/N)**	4.8% (4/83)	8.1% (7/86)	6.5% (11/169)	0.3817 (#)
	**Hispanic**	**% (n/N)**	10.8% (9/83)	4.7% (4/86)	7.7% (13/169)	0.1310 (#)
	**Other**	**% (n/N)**	3.6% (3/83)	8.1% (7/86)	5.9% (10/169)	0.2126 (#)
**Education**	**< 9 years of education**	**% (n/N)**	2.4% (2/83)	-	1.2% (2/169)	**0.0332** (#)
	**9 to 12 years of education**	**% (n/N)**	26.5% (22/83)	11.6% (10/86)	18.9% (32/169)	
	**Over 12 years of education**	**% (n/N)**	69.9% (58/83)	86.0% (74/86)	78.1% (132/169)	
	**Unknown**	**% (n/N)**	1.2% (1/83)	2.3% (2/86)	1.8% (3/169)	
**Age**	**Years**	**N**	83	86	169	0.2969 (*)
		**Mean (SD)**	46.3 (11.32)	44.4 (12.00)	45.4 (11.67)	
		**Median [Range]**	47.0 [22.1; 65.8]	44.9 [22.5; 68.9]	46.6 [22.1; 68.9]	
**First episode**	**Age**	**N**	83	86	169	0.1972 (*)
		**Mean (SD)**	25.2 (12.28)	22.8 (12.44)	24.0 (12.38)	
		**Median [Range]**	23.0 [5; 63]	18.5 [0; 54]	20.0 [0; 63]	
		**N**	83	85	168	0.1228 (*)

**Current episode**	**Duration (months)**	**Mean (SD)**	17.6 (14.69)	21.3 (16.01)	19.5 (15.44)	
		**Median [Range]**	12.0 [1; 60]	16.0 [2; 60]	13.0 [1; 60]	
**Previous episodes**	**Number**	**N**	83	85	168	0.5406 (*)
		**Mean (SD)**	10.0 (20.80)	8.2 (15.84)	9.1 (18.42)	
		**Median [Range]**	4.0 [0; 99]	4.0 [0; 99]	4.0 [0; 99]	
**Depression related hospitalizations**	**Incidences**	**N**	83	86	169	0.5120 (*)
		**Mean (SD)**	0.6 (1.83)	0.5 (1.32)	0.6 (1.59)	
		**Median [Range]**	0.0 [0; 15]	0.0 [0; 10]	0.0 [0; 15]	
**Anti-depressive agents**	**Current episode**	**N**	83	86	169	0.3798 (*)
		**Mean (SD)**	2.8 (1.84)	3.1 (2.02)	3.0 (1.93)	
		**Median [Range]**	2.0 [1; 9]	3.0 [1; 12]	2.0 [1; 12]	
**Suicide attempts**	**Incidences**	**% (n/N)**	13.3% (11/83)	18.6% (16/86)	16.0% (27/169)	0.3425 (#)

(*) t-test; (#) Chi-square test

Table S3. Baseline Antidepressant Treatment History Form (ATHF) (ITT)

			H1 Coil	H7 Coil	All	p-value
# of medications in the highest ATHF level	1	% (n/N)	72.3% (60/83)	75.6% (65/86)	74.0% (125/169)	0.6632 (*)
	2	% (n/N)	18.1% (15/83)	17.4% (15/86)	17.8% (30/169)	
	3	% (n/N)	7.2% (6/83)	5.8% (5/86)	6.5% (11/169)	
	4	% (n/N)	1.2% (1/83)	-	0.6% (1/169)	
	5	% (n/N)	-	1.2% (1/86)	0.6% (1/169)	
	9	% (n/N)	1.2% (1/83)	-	0.6% (1/169)	
Highest AD resistance level	1	% (n/N)	1.2% (1/83)	4.7% (4/86)	3.0% (5/169)	0.6642 (*)
	2	% (n/N)	7.2% (6/83)	5.8% (5/86)	6.5% (11/169)	
	3	% (n/N)	41.0% (34/83)	45.3% (39/86)	43.2% (73/169)	
	4	% (n/N)	48.2% (40/83)	41.9% (36/86)	45.0% (76/169)	
	5	% (n/N)	2.4% (2/83)	2.3% (2/86)	2.4% (4/169)	

(*) t-test

Table S4. Baseline clinical characteristics (ITT)

Baseline Visit		H1 Coil	H7 Coil	All	p-value
**HARS**	**N**	82	86	168	0.3919 (*)
	**Mean (SD)**	19.9 (6.53)	19.1 (5.88)	19.5 (6.21)	
	**Median [Range]**	19.0 [6; 43]	19.0 [9; 36]	19.0 [6; 43]	
**QIDS-SR**	**N**	82	85	167	0.7251 (*)
	**Mean (SD)**	18.6 (5.64)	19.0 (5.60)	18.8 (5.61)	
	**Median [Range]**	20.0 [6; 33]	19.0 [7; 35]	19.0 [6; 35]	
**CGI-I**	**N**	83	86	169	0.5580 (*)
	**Mean (SD)**	5.1 (0.46)	5.2 (0.47)	5.2 (0.46)	
	**Median [Range]**	5.0 [4; 7]	5.0 [4; 7]	5.0 [4; 7]	
**PGI-I**	**N**	82	85	167	0.8847 (*)
	**Mean (SD)**	4.2 (0.74)	4.2 (0.67)	4.2 (0.70)	
	**Median [Range]**	4.0 [3; 7]	4.0 [2; 6]	4.0 [2; 7]	
**GAF**	**N**	82	86	168	0.6403 (*)
	**Mean (SD)**	50.4 (8.20)	51.0 (8.62)	50.7 (8.40)	
	**Median [Range]**	51.0 [21; 75]	51.0 [21; 70]	51.0 [21; 75]	
**Q-LES-Q**	**N**	82	84	166	0.3142 (*)
	**Mean (SD)**	37.96 (14.98)	35.69 (13.91)	36.81 (14.45)	
	**Median [Range]**	38.39 [7.1; 75]	35.71 [3.6; 71.4]	35.71 [3.6; 75]	

(*) t-test

Table S5. Descriptive Statistics of HDRS scores – ITT set

HDRS-21		H1 Coil		H7 Coil	
		**Total Score**	**Change from Baseline**	**Total Score**	**Change from Baseline**
**Baseline Visit**	**N**	83		86	
	**Mean (SD)**	24.2 (3.57)		23.8 (3.41)	
	**Median [Range]**	23.0 [20; 36]		24.0 [19; 34]	
**3 Week Visit**	**N**	69	69	81	81
	**Mean (SD)**	16.7 (5.25)	-7.3 (5.05)	16.5 (5.13)	-7.5 (5.27)
	**Median [Range]**	17.0 [3; 30]	-7.0 [-25; 4]	17.0 [7; 28]	-7.0 [-22; 5]
**4 Week Visit**	**N**	67	67	81	81
	**Mean (SD)**	14.0 (5.16)	-9.9 (5.50)	13.7 (5.91)	-10.2 (5.98)
	**Median [Range]**	15.0 [3; 25]	-8.0 [-23; -1]	14.0 [1; 29]	-10.0 [-25; 3]
**5 Week Visit**	**N**	65	65	81	81
	**Mean (SD)**	12.4 (5.32)	-11.4 (5.82)	12.2 (5.86)	-11.8 (6.28)
	**Median [Range]**	13.0 [1; 24]	-11.0 [-26; 1]	11.0 [0; 25]	-11.0 [-26; 4]
**6 Week Visit**	**N**	64	64	81	81
	**Mean (SD)**	10.8 (6.20)	-12.9 (6.84)	10.9 (6.62)	-13.0 (6.83)
	**Median [Range]**	10.0 [1; 27]	-14.0 [-30; 6]	10.0 [0; 26]	-13.0 [-28; 4]

Table S6. Descriptive Statistics of HDRS scores – CO set

HDRS-21		H1 Coil		H7 Coil	
		Total Score	Change from Baseline	Total Score	Change from Baseline
Baseline Visit	N	64		79	
	Mean (SD)	23.7 (3.08)		23.9 (3.51)	
	Median [Range]	23.0 [20;33]		24.0 [19; 34]	
3 Week Visit	N	64	64	79	79
	Mean (SD)	16.5 (5.23)	-7.2 (4.98)	16.4 (5.14)	-7.6 (5.28)
	Median [Range]	16.5 [3;30]	-7.0 [-25; 4]	17.0 [7; 28]	-7.0 [-22; 5]
4 Week Visit	N	64	64	79	79
	Mean (SD)	13.9 (5.14)	-9.8 (5.33)	13.6 (5.87)	-10.4 (5.95)
	Median [Range]	14.5 [3;25]	-8.0 [-21; -1]	13.0 [1; 29]	-11.0 [-25; 3]
5 Week Visit	N	64	64	79	79
	Mean (SD)	12.5 (5.30)	-11.2 (5.57)	12.1 (5.92)	-11.8 (6.35)
	Median [Range]	13.0 [1;24]	-11.0 [-24; 1]	11.0 [0; 25]	-11.0 [-26; 4]
6 Week Visit	N	64	64	79	79
	Mean (SD)	10.8 (6.20)	-12.9 (6.84)	10.8 (6.61)	-13.1 (6.84)
	Median [Range]	10.0 [1;27]	-14.0 [-30; 6]	10.0 [0; 26]	-13.0 [-28; 4]

Table S7. Adjusted means of HDRS-21 changes by week – ITT set

HDRS-21			Estimate	SE	p-value	95% CI
3 Week Visit	Adjusted means of the changes	H1 Coil	-7.096	0.637	< 0.0001	[-8.355; -5.836]
		H7 Coil	-7.137	0.606	< 0.0001	[-8.335; -5.940]
	Comparison of the adjusted means	Diff (H7-H1)	-0.041	0.766	0.9569	[-1.556; 1.473]
4 Week Visit	Adjusted means of the changes	H1 Coil	-9.679	0.724	< 0.0001	[-11.110; -8.248]
		H7 Coil	-9.878	0.68	< 0.0001	[-11.221; -8.535]
	Comparison of the adjusted means	Diff (H7-H1)	-0.199	0.895	0.8244	[-1.969; 1.571]
5 Week Visit	Adjusted means of the changes	H1 Coil	-11.16	0.7	< 0.0001	[-12.543; -9.776]
		H7 Coil	-11.446	0.654	< 0.0001	[-12.738; -10.154]
	Comparison of the adjusted means	Diff (H7-H1)	-0.286	0.855	0.7382	[-1.977; 1.404]
6 Week Visit	Adjusted means of the changes	H1 Coil	-12.875	0.813	< 0.0001	[-14.481; -11.268]
		H7 Coil	-12.646	0.752	< 0.0001	[-14.132; -11.160]
	Comparison of the adjusted means	Diff (H7-H1)	0.229	1.02	0.8231	[-1.789; 2.246]

Table S8. Adjusted means of HDRS-21 changes by week – CO set

HDRS-21			Estimate	SE	p-value	95% CI
**3 Week Visit**	**Adjusted means of the changes**	**H1 Coil**	-7.098	0.669	< 0.0001	[-8.421; -5.775]
		**H7 Coil**	-7.231	0.631	< 0.0001	[-8.478; -5.984]
	**Comparison of the adjusted means**	**Diff (H7-H1)**	-0.133	0.794	0.8676	[-1.704; 1.439]
**4 Week Visit**	**Adjusted means of the changes**	**H1 Coil**	-9.629	0.75	< 0.0001	[-11.113; -8.146]
		**H7 Coil**	-10.041	0.701	< 0.0001	[-11.426; -8.656]
	**Comparison of the adjusted means**	**Diff (H7-H1)**	-0.412	0.917	0.6542	[-2.225; 1.402]
**5 Week Visit**	**Adjusted means of the changes**	**H1 Coil**	-11.067	0.724	< 0.0001	[-12.497; -9.637]
		**H7 Coil**	-11.497	0.678	< 0.0001	[-12.836; -10.157]
	**Comparison of the adjusted means**	**Diff (H7-H1)**	-0.43	0.877	0.6248	[-2.164; 1.305]
**6 Week Visit**	**Adjusted means of the changes**	**H1 Coil**	-12.801	0.833	< 0.0001	[-14.447; -11.155]
		**H7 Coil**	-12.775	0.773	< 0.0001	[-14.302; -11.248]
	**Comparison of the adjusted means**	**Diff (H7-H1)**	0.026	1.038	0.9799	[-2.026; 2.079]

Table S9. Adjusted means of HDRS-21 changes from baseline to week 6

		Estimate	SD	Pr > |t|	95% CI	Upper limit of the one-sided 95% CI
**ITT**	**H1 Coil**	-12.875	0.813	< 0.0001	[-14.481; -11.268]	
	**H7 Coil**	-12.646	0.752	< 0.0001	[-14.132; -11.160]	
	**Diff (H7-H1)**	0.229	1.021	0.8231	[-1.789; 2.246]	**1.918**
**CO**	**H1 Coil**	-12.801	0.833	< 0.0001	[-14.447; -11.155]	
	**H7 Coil**	-12.775	0.773	< 0.0001	[-14.302; -11.248]	
	**Diff (H7-H1)**	0.026	1.038	0.9799	[-2.026; 2.079]	**1.745**

Table S10. Descriptive Statistics of the Safety Parameters by Visit - ITT

			H1 Coil		H7 Coil	
			Value	Change from Baseline	Value	Change from Baseline
YMRS	Baseline Visit	N	82		86	
		Mean (SD)	1.2 (1.94)		1.5 (2.31)	
		Median [Range]	0.0 [0; 13]		0.0 [0; 13]	
	6 Week Visit	N	64	64	80	80
		Mean (SD)	0.8 (1.21)	-0.2 (1.45)	1.1 (1.71)	-0.4 (2.07)
		Median [Range]	0.0 [5;0]	0.0 [3;4-]	0.0 [8;0]	0.0 [5;12-]
SSI	Screening Visit	N	82		85	
		Mean (SD)	1.4 (3.16)		2.4 (4.66)	
		Median [Range]	0.0 [0; 13]		0.0 [0; 25]	
	Baseline Visit	N	83		86	
		Mean (SD)	0.9 (2.57)		2.2 (4.12)	
		Median [Range]	0.0 [0; 12]		0.0 [0; 20]	
	6 Week Visit	N	64	64	79	79
		Mean (SD)	0.6 (1.80)	-0.5 (2.75)	0.9 (2.43)	-1.1 (3.07)
		Median [Range]	0.0 [7;0]	0.0 [7;12-]	0.0 [11;0]	0.0 [6;11-]
MMSE	Screening Visit	N	83		85	
		Mean (SD)	29.3 (1.03)		29.2 (0.98)	
		Median [Range]	30.0 [26; 30]		30.0 [26; 30]	
	6 Week Visit	N	63	63	80	79
		Mean (SD)	29.0 (1.16)	-0.4 (1.09)	29.4 (0.89)	0.1 (1.05)
		Median [Range]	29.0 [30;26]	0.0 [2;3-]	30.0 [30;27]	0.0 [3;3-]
		N	80		86	

**BSRT - LTS Percentile**	**Baseline Visit**	**Mean (SD)**	31.4 (29.65)		38.5 (30.82)	
		**Median [Range]**	25.0 [0; 100]		25.0 [0; 100]	
	**6 Week Visit**	**N**	63	63	80	80
		**Mean (SD)**	37.0 (30.39)	10.9 (26.80)	46.3 (33.18)	8.7 (30.54)
		**Median [Range]**	25.0 [100;0]	0.0 [95;50-]	50.0 [100;0]	0.0 [95;50-]
**BSRT - CLTR Percentile**	**Baseline Visit**	**N**	80		86	
		**Mean (SD)**	28.3 (27.52)		35.6 (31.08)	
		**Median [Range]**	25.0 [0; 100]		25.0 [0; 100]	
	**6 Week Visit**	**N**	63	63	80	80
		**Mean (SD)**	33.5 (29.68)	8.9 (25.72)	40.5 (31.59)	4.9 (34.71)
		**Median [Range]**	25.0 [100;0]	0.0 [95;45-]	37.5 [100;0]	0.0 [95;95-]
**BSRT - Total Number of Intrusions**	**Baseline Visit**	**N**	80		86	
		**Mean (SD)**	0.8 (1.48)		1.2 (1.86)	
		**Median [Range]**	0.0 [0; 7]		0.5 [0; 10]	
	**6 Week Visit**	**N**	63	63	80	80
		**Mean (SD)**	1.2 (2.41)	0.3 (2.38)	1.0 (1.86)	-0.3 (1.75)
		**Median [Range]**	0.0 [13;0]	0.0 [12;6-]	0.0 [8;0]	0.0 [6;5-]
**BSRT - Total Number of Words at Delayed Recall**	**Baseline Visit**	**N**	80		86	
		**Mean (SD)**	6.6 (3.02)		7.1 (3.28)	
		**Median [Range]**	7.0 [0; 12]		7.0 [0; 12]	
	**6 Week Visit**	**N**	63	63	80	80
		**Mean (SD)**	6.5 (3.21)	0.0 (2.23)	6.7 (3.68)	-0.3 (2.99)
		**Median [Range]**	6.0 [12;0]	0.0 [8;7-]	7.0 [12;0]	0.0 [9;9-]

Table S11. Adverse Event - ITT

		H1			H7			
		Reports	Rate	Incidence	Reports	Rate	Incidence	P-value*
**Total**		374	4.51	72.29%	369	4.29	75.58%	0.7263
**Cardiac disorders**	**Total**	.	.	.	1	0.01	1.16%	1
	**Palpitations**	.	.	.	1	0.01	1.16%	1
**Eye disorders**	**Total**	2	0.02	2.41%	4	0.05	4.65%	0.6821
	**Eye pain**	1	0.01	1.20%	.	.	.	0.4911
	**Eyes tearing**	.	.	.	1	0.01	1.16%	1
	**Swollen eyelid**	1	0.01	1.20%	.	.	.	0.4911
	**Vision blurred**	.	.	.	3	0.03	3.49%	0.2458
**Gastrointestinal disorders**	**Total**	31	0.37	16.87%	24	0.28	17.44%	1
	**Abdominal discomfort**	.	.	.	2	0.02	2.33%	0.4972
	**Abdominal pain**	2	0.02	2.41%	3	0.03	3.49%	1
	**Abdominal pain upper**	.	.	.	1	0.01	1.16%	1
	**Dental pain**	6	0.07	2.41%	4	0.05	2.33%	1
	**Diarrhea**	.	.	.	1	0.01	1.16%	1
	**Dyspepsia**	1	0.01	1.20%	1	0.01	1.16%	1
	**Food poisoning**	2	0.02	2.41%	.	.	.	0.2397
	**Gastrointestinal disorder**	7	0.08	2.41%	1	0.01	1.16%	0.6161
	**Nausea**	11	0.13	8.43%	8	0.09	6.98%	0.779
	**Tooth ache**	.	.	.	1	0.01	1.16%	1
	**Vomiting**	2	0.02	2.41%	2	0.02	2.33%	1
**General disorders and administration site conditions**	**Total**	52	0.63	31.33%	55	0.64	37.21%	0.5171
	**Application site discomfort**	34	0.41	20.48%	33	0.38	20.93%	1
	**Application site pain**	11	0.13	8.43%	14	0.16	13.95%	0.3319

	**Discomfort**	1	0.01	1.20%	1	0.01	1.16%	1
	**Fatigue**	6	0.07	4.82%	4	0.05	2.33%	0.4378
	**Pain**	.	.	.	1	0.01	1.16%	1
	**Pain in face**	.	.	.	1	0.01	1.16%	1
	**Pyrexia**	.	.	.	1	0.01	1.16%	1
**Immune system disorders**	**Total**	2	0.02	1.20%	3	0.03	3.49%	0.6207
	**Allergic sinusitis**	2	0.02	1.20%	2	0.02	2.33%	1
	**Hypersensitivity**	.	.	.	1	0.01	1.16%	1
**Infections and infestations**	**Total**	14	0.17	10.84%	17	0.2	11.63%	1
	**Abscess oral**	1	0.01	1.20%	.	.	.	0.4911
	**Hordeolum**	1	0.01	1.20%	.	.	.	0.4911
	**Influenza**	2	0.02	2.41%	1	0.01	1.16%	0.6161
	**Nasopharyngitis**	6	0.07	6.02%	10	0.12	8.14%	0.7664
	**Onychomycosis**	.	.	.	1	0.01	1.16%	1
	**Otitis externa**	1	0.01	1.20%	.	.	.	0.4911
	**Pharyngitis**	1	0.01	1.20%	3	0.03	3.49%	0.6207
	**Pharyngitis streptococcal**	.	.	.	2	0.02	2.33%	0.4972
	**Streptococcal tonsillitis**	1	0.01	1.20%	.	.	.	0.4911
	**Tonsillitis streptococcal**	1	0.01	1.20%	.	.	.	0.4911
**Metabolism and nutrition disorders**	**Total**	.	.	.	1	0.01	1.16%	1
	**Dehydration**	.	.	.	1	0.01	1.16%	1
**Musculoskeletal and connective tissue disorders**	**Total**	71	0.86	30.12%	40	0.47	26.74%	0.7332
	**Arthralgia**	5	0.06	4.82%	8	0.09	6.98%	0.7467
	**Back pain**	7	0.08	7.23%	8	0.09	9.30%	0.7818
	**Crackling jaw**	1	0.01	1.20%	.	.	.	0.4911
	**Facet joint syndrome**	7	0.08	1.20%	.	.	.	0.4911
	**Muscle spasms**	1	0.01	1.20%	1	0.01	1.16%	1

	**Muscle strain**	.	.	.	1	0.01	1.16%	1
	**Muscle twitching**	25	0.3	6.02%	3	0.03	3.49%	0.4908
	**Muscular weakness**	1	0.01	1.20%	.	.	.	0.4911
	**Musculoskeletal discomfort**	1	0.01	1.20%	3	0.03	3.49%	0.6207
	**Myalgia**	4	0.05	3.61%	5	0.06	3.49%	1
	**Neck pain**	12	0.14	9.64%	7	0.08	6.98%	0.586
	**Pain in extremity**	1	0.01	1.20%	1	0.01	1.16%	1
	**Pain in jaw**	4	0.05	3.61%	2	0.02	2.33%	0.6782
	**Sciatica**	1	0.01	1.20%	.	.	.	0.4911
	**Temporomandibular joint syndrome**	1	0.01	1.20%	.	.	.	0.4911
	**Trismus**	.	.	.	1	0.01	1.16%	1
**Nervous system disorders**	**Total**	171	2.06	51.81%	185	2.15	52.33%	1
	**Dizziness**	7	0.08	6.02%	8	0.09	6.98%	1
	**Headache**	150	1.81	46.99%	161	1.87	50.00%	0.7589
	**Migraine**	5	0.06	4.82%	11	0.13	5.81%	1
	**Paresthesia**	2	0.02	2.41%	2	0.02	1.16%	0.6161
	**Restlessness**	3	0.04	2.41%	1	0.01	1.16%	0.6161
	**Tinnitus**	4	0.05	1.20%	.	.	.	0.4911
	**Vertigo**	.	.	.	2	0.02	2.33%	0.4972
**Psychiatric disorders**	**Total**	25	0.3	20.48%	26	0.3	18.60%	0.8468
	**Abnormal dreams**	.	.	.	2	0.02	2.33%	0.4972
	**Agitation**	1	0.01	1.20%	1	0.01	1.16%	1
	**Anxiety**	5	0.06	4.82%	5	0.06	5.81%	1
	**Aphasia**	1	0.01	1.20%	.	.	.	0.4911
	**Bruxism**	3	0.04	2.41%	3	0.03	3.49%	1
	**Claustrophobia**	.	.	.	1	0.01	1.16%	1

	**Depression**	1	0.01	1.20%	.	.	.	0.4911
	**Disorientation**	1	0.01	1.20%	1	0.01	1.16%	1
	**Frustration tolerance decreased**	.	.	.	2	0.02	1.16%	1
	**Hypersomnia**	.	.	.	1	0.01	1.16%	1
	**Insomnia**	7	0.08	7.23%	7	0.08	5.81%	0.7635
	**Nightmare**	1	0.01	1.20%	1	0.01	1.16%	1
	**Panic attacks**	2	0.02	2.41%	.	.	.	0.2397
	**Poor quality sleep**	.	.	.	2	0.02	1.16%	1
	**Sleep disorder**	1	0.01	1.20%	.	.	.	0.4911
	**Suicidal ideation**	1	0.01	1.20%	.	.	.	0.4911
	**Suicide attempt**	1	0.01	1.20%	.	.	.	0.4911
**Reproductive system and breast disorders**	**Total**	2	0.02	2.41%	5	0.06	5.81%	0.4437
	**Dysmenorrhea**	2	0.02	2.41%	2	0.02	2.33%	1
	**Hot flush**	.	.	.	1	0.01	1.16%	1
	**Menstrual irregular**	.	.	.	1	0.01	1.16%	1
	**Ovarian cyst ruptured**	.	.	.	1	0.01	1.16%	1
**Respiratory, thoracic and mediastinal disorders**	**Total**	3	0.04	3.61%	8	0.09	4.65%	1
	**Cough**	.	.	.	2	0.02	2.33%	0.4972
	**Nasal congestion**	.	.	.	1	0.01	1.16%	1
	**Rhinorrhea**	2	0.02	2.41%	4	0.05	3.49%	1
	**Upper respiratory tract infection**	1	0.01	1.20%	1	0.01	1.16%	1
**Skin and subcutaneous tissue disorders**	**Total**	1	0.01	1.20%	.	.	.	0.4911
	**Pruritus**	1	0.01	1.20%	.	.	.	0.4911

*P-value - Fisher's exact test

References

1. Alyagon U, Shahar H, Hadar A, Barnea-Ygael N, Lazarovits A, Shalev H, and Zangen A. Alleviation of ADHD symptoms by non-invasive right prefrontal stimulation is correlated with EEG activity. *NeuroImage: Clinical.* 2020;26(102206.

2. Allen EA, Pasley BN, Duong T, and Freeman RD. Transcranial Magnetic Stimulation Elicits Coupled Neural and Hemodynamic Consequences. *Science.* 2007;317(5846):1918-21.

3. Jensen O, and Mazaheri A. Shaping Functional Architecture by Oscillatory Alpha Activity: Gating by Inhibition. *Front Hum Neurosci.* 2010;4(

4. Muthukumaraswamy S. High-frequency brain activity and muscle artifacts in MEG/EEG: a review and recommendations. *Front Hum Neurosci.* 2013;7(138.

5. Chekroud AM, Gueorguieva R, Krumholz HM, Trivedi MH, Krystal JH, and McCarthy G. Reevaluating the Efficacy and Predictability of Antidepressant Treatments: A Symptom Clustering Approach. *JAMA Psychiatry.* 2017;74(4):370-8.

6. O'Reardon JP, Solvason HB, Janicak PG, Sampson S, Isenberg KE, Nahas Z, McDonald WM, Avery D, Fitzgerald PB, and Loo C. Efficacy and safety of transcranial magnetic stimulation in the acute treatment of major depression: a multisite randomized controlled trial. *Biological psychiatry.* 2007;62(11):1208-16.

7. Shafer AB. Meta-analysis of the factor structures of four depression questionnaires: Beck, CES-D, Hamilton, and Zung. *Journal of Clinical Psychology.* 2006;62(1):123-46.

8. Brakemeier E-L, Luborzewski A, Danker-Hopfe H, Kathmann N, and Bajbouj M. Positive predictors for antidepressive response to prefrontal repetitive transcranial magnetic stimulation (rTMS). *Journal of Psychiatric Research.* 2007;41(5):395-403.

9. Fava M, Rush AJ, Alpert JE, Balasubramani GK, Wisniewski SR, Carmin CN, Biggs MM, Zisook S, Leuchter A, Howland R, et al. Difference in Treatment Outcome in Outpatients With Anxious Versus Nonanxious Depression: A STAR*D Report. *American Journal of Psychiatry.* 2008;165(3):342-51.

10. van der Knaap LJ, and van der Ham IJ. How does the corpus callosum mediate interhemispheric transfer? A review. *Behavioural brain research.* 2011;223(1):211-21.

11. Palmer LM, Schulz JM, Murphy SC, Ledergerber D, Murayama M, and Larkum ME. The cellular basis of GABAB-mediated interhemispheric inhibition. *Science.* 2012;335(6071):989-93.

12. Hagemann D, Naumann E, and Thayer JF. The quest for the EEG reference revisited: A glance from brain asymmetry research. *Psychophysiology.* 2001;38(5):847-57.

13. Ge R, Downar J, Blumberger DM, Daskalakis ZJ, Lam RW, and Vila-Rodriguez F. Structural network integrity of the central executive network is associated with the therapeutic effect of rTMS in treatment resistant depression. *Progress in Neuro-Psychopharmacology and Biological Psychiatry.* 2019;92(217-25.

14. Liston C, Chen AC, Zebley BD, Drysdale AT, Gordon R, Leuchter B, Voss HU, Casey BJ, Etkin A, and Dubin MJ. Default Mode Network Mechanisms of Transcranial Magnetic Stimulation in Depression. *Biological Psychiatry.* 2014;76(7):517-26.

15. Zhou H-X, Chen X, Shen Y-Q, Li L, Chen N-X, Zhu Z-C, Castellanos FX, and Yan C-G. Rumination and the default mode network: Meta-analysis of brain imaging studies and implications for depression. *NeuroImage.* 2020;206(116287.

16. Mak LE, Minuzzi L, MacQueen G, Hall G, Kennedy SH, and Milev R. The default mode network in healthy individuals: a systematic review and meta-analysis. *Brain connectivity.* 2017;7(1):25-33.

17. Seeley WW, Menon V, Schatzberg AF, Keller J, Glover GH, Kenna H, Reiss AL, and Greicius MD. Dissociable intrinsic connectivity networks for salience processing and executive control. *Journal of Neuroscience.* 2007;27(9):2349-56.

18. Fox MD, Snyder AZ, Vincent JL, Corbetta M, Van Essen DC, and Raichle ME. The human brain is intrinsically organized into dynamic, anticorrelated functional networks. *Proceedings of the National Academy of Sciences.* 2005;102(27):9673-8.

19. Li J, Liu J, Zhong Y, Wang H, Yan B, Zheng K, Wei L, Lu H, and Li B. Causal Interactions Between the Default Mode Network and Central Executive Network in Patients with Major Depression. *Neuroscience.* 2021;475(93-102.

20. Chen AC, Oathes DJ, Chang C, Bradley T, Zhou Z-W, Williams LM, Glover GH, Deisseroth K, and Etkin A. Causal interactions between fronto-parietal central executive and default-mode networks in humans. *Proceedings of the National Academy of Sciences.* 2013;110(49):19944-9.

21. Jung T-P, Makeig S, Humphries C, Lee T-W, McKeown MJ, Iragui V, and Sejnowski TJ. Removing electroencephalographic artifacts by blind source separation. *Psychophysiology.* 2000;37(2):163-78.

22. Tendler A, Sisko E, Barnea-Ygael N, DeLuca M, Rodriguez N, Corbett-Methott S, Sutton J, and Zangen A. Antidepressant remission to dTMS of the dmPFC and ACC in lateral PFC dTMS nonresponders: Case series. *Brain stimulation.* 2017;10(3):714-5.

23. Levkovitz Y, Isserles M, Padberg F, Lisanby SH, Bystritsky A, Xia G, Tendler A, Daskalakis ZJ, Winston JL, and Dannon P. Efficacy and safety of deep transcranial magnetic stimulation for major depression: a prospective multicenter randomized controlled trial. *World Psychiatry.* 2015;14(1):64-73.

24. Jakobsen JC, Gluud C, and Kirsch I. Should antidepressants be used for major depressive disorder? *BMJ evidence-based medicine.* 2020;25(4):130-.
